# Diversity and taxonomy of the genus *Amanita* (Amanitaceae, Agaricales) in the Yanshan Mountains, Northern China

**DOI:** 10.3389/fpls.2023.1226794

**Published:** 2023-09-14

**Authors:** Hao Zhou, MeiJun Guo, Lan Zhuo, HuiFang Yan, XiaoNan Sui, Yue Gao, ChengLin Hou

**Affiliations:** College of Life Science, Capital Normal University, Beijing, China

**Keywords:** new taxa, poisonous fungi, ectomycorrhizal, species diversity, molecular systematics

## Abstract

Globally, the species of *Amanita* are key components of ectomycorrhizal ecosystems. Some of them are widely known as poisonous or edible fungi. Although many new *Amanita* species from China have been described, the species diversity of Yanshan Mountains remains unknown. We here describe three new species, namely, *A. borealis* sp. nov. (Sect. *Amanita*), *A. brunneola* sp. nov. (Sect. *Caesareae*), and *A. yanshanensis* sp. nov. (Sect. *Validae*), based on morphological observations and molecular phylogenetic analyses. In addition, nine known species, namely, *A. caesareoides* (Sect. *Caesareae*), *A. chiui* (Sect. *Vaginatae*), *A. muscaria* (Sect. *Amanita*), *A. oberwinklerana* (Sect. *Roanokenses*), *A. ovalispora* (Sect. *Vaginatae*), *A. subglobosa* (Sect. *Amanita*), *A. subjunquillea* (Sect. *phalloideae*), *A. vaginata* var. *vaginata* (Sect. *Vaginatae*), and *A. virosa* (Sect. *phalloideae*), were reported from Yanshan Mountains for the first time. Our results emphasize that China has a high diversity of *Amanita* species and that additional studies are required to understand the exact species number. These findings play a crucial role in *Amanita* toxin research and ecological conservation. This study investigated the areas where *Amanita* species-related research is lacking. The study also attempted to better understand *Amanita* distribution and thus contribute to related research. This study enriches the species diversity of *Amanita* in Yanshan Mountains and offers additional data supporting the macrofungal systematics, toxin research, and diversity and ecological studies of *Amanita* in future studies.

## 1 Introduction

*Amanita* Pers., the largest genus of the family *Amanitaceae* E.-J. Gilbert, was established by Persoon (Persoon, 1797). It is an almost cosmopolitan genus comprising approximately 650 accepted species (Yang et al., 2004; Tulloss, 2005; Yang, 2005; Menolli et al., 2009; Yang, 2015; Kim et al., 2013a, b; Cai et al., 2014; Ariyawansa et al., 2015; Cho et al., 2015; Cai et al., 2016; Cui et al., 2018; Yang et al., 2018; Fraiture et al., 2019; Mighell et al., 2019; Mighell et al., 2021; Su et al., 2022).

Most *Amanita* species are ectomycorrhizal fungi of ecological importance, and more than 10 plant families are known to be symbiotically associated with *Amanita* (Beeli et al., 1935; Reid, 1980; Pegler and Shah-Smith, 1997; Wood, 1997; Yang, 1997; Yang, 2005; Davison et al., 2017; Cui et al., 2018). However, some *Amanita* species may be saprotrophic (e.g., *A. pruittii* A. H. Sm. ex Tulloss) (Cui et al., 2018).

Some species are commonly known as edible fungi, such as *A. caesarea* (Scop.) Pers., *A. sinensis* Zhu L. Yang, and *A. yuaniana* Zhu L. Yang et al. In addition, some *Amanita* species are poisonous, including *A. subjunquillea* S. Imai, *A. virosa* Bertill., and *A. tenuifolia* (Murrill) Murrill et al. (Yang, 2005). In China, deaths caused by consuming poisonous *Amanita* species are common (Li et al., 2020; Li et al., 2021a; Li et al., 2021b).

Based on the traditional morphological and anatomical characteristics, and the molecular phylogeny evidence, the classification of *Amanita* has also undergone many changes. Corner and Bas (1962) and Bas (1969) considered observing the natural characteristics of *Amanita* species in the wild important. They applied microscopic characteristics to taxonomy and split the species into two subgenera and six sections. Many mycologists have accepted this taxonomic method as a great historical advance in the *Amanita* classification (Jenkins, 1977; Hongo, 1982; Jenkins, 1986; Mao, 1990; Pegler and Shah-Smith, 1997). However, the classification of its subgenera remains disputed (Moser, 1967; Garcin, 1984; Singer, 1986). Subsequently, the systematic research on *Amanita* is gradually deepening with the development and advancement of molecular systematics. A recent comprehensive phylogenetic study introduced the latest classification system for *Amanita.* According to this system, *Amanita* was divided into 3 subgenera and 11 sections (Cui et al., 2018). This system is followed by other mycologists (Kumar et al., 2021; Suwannarach et al., 2022; Huang et al., 2023).

**Figure 1:**
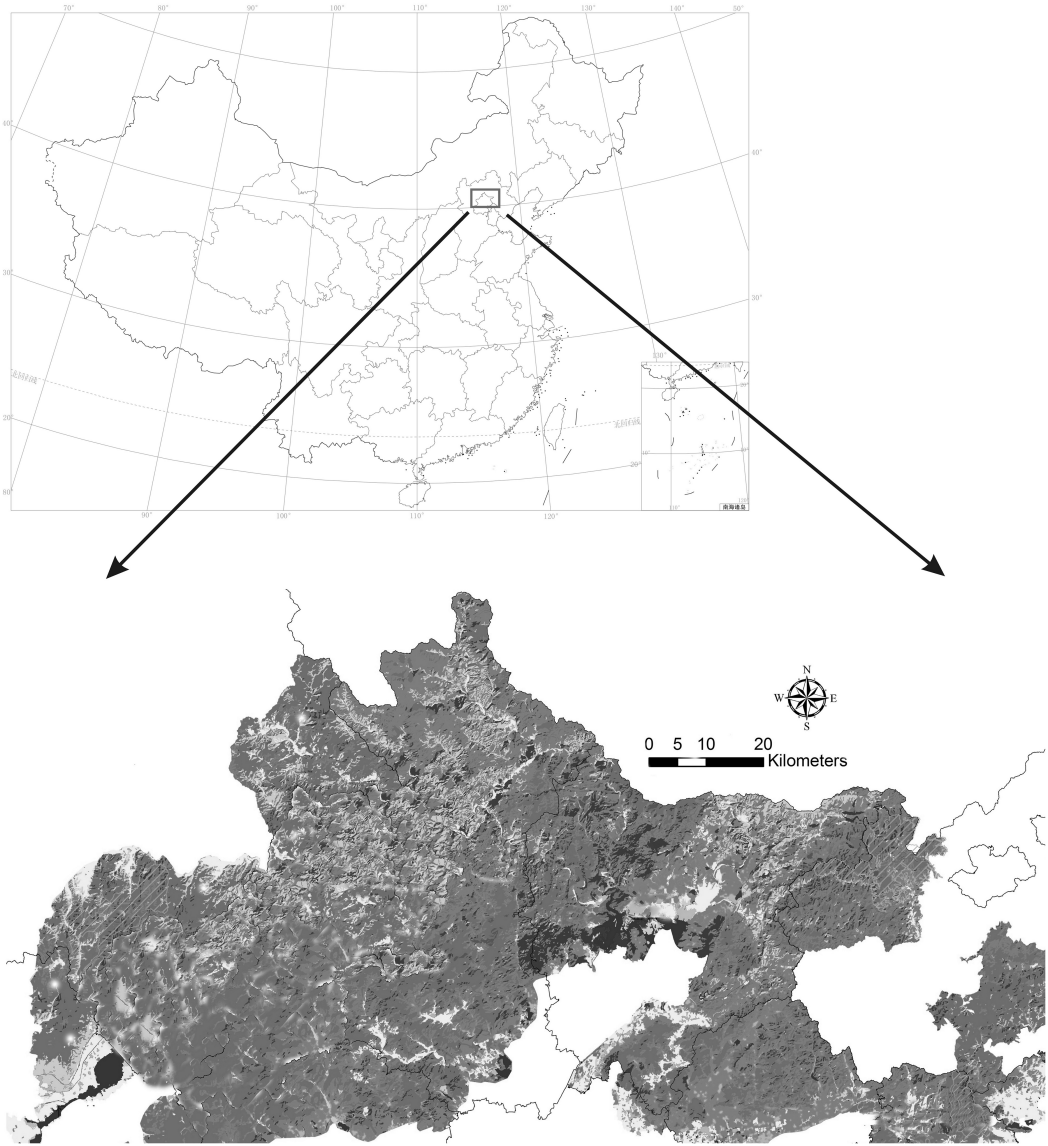
Geographical location and topography of the study area.

The Yanshan Mountains (115°–119°47′E, 39°40′–41°20′N) is located in northern China and has a high plant diversity (**Figure 1**). The main forest types on these mountains are deciduous broad-leaved forests and mixed coniferous and broad-leaved forests. The original dominant ectomycorrhizal plants included *Quercus mongolica* Fisch. ex Ledeb., *Betula platyphylla* Suk., *Abies nephrolepis* (Trautv.) Maxim., *Populus tomentosa* Carrière, and *Pinus tabuliformis* Carr. (Wang et al., 2021). The Yanshan Mountains region has a warm–temperate continental monsoon climate with an annual precipitation of 350–700 mm. The peak of precipitation occurs in June–August. The altitude of these mountains ranges from 200 to 2,200 m (Zhou et al., 2022a; Zhou et al., 2022b; Zhou et al., 2022c). Some past records of *Amanita* species in this area are available (Yang, 2004; Chen et al., 2006; Zhang et al., 2017; Cui et al., 2018; Wu et al., 2020). However, information available on *Amanita* species on these mountains is incomplete.

Taxonomic research has never been considered a popular study, but it can be considered the basis for understanding the world and research in related professional fields and can only be studied and applied if we figure out what the species really is. Especially think of *Amanita*, which are a very attractive taxa of macrofungi. It plays an important role in toxin research and ecological conservation. In the present study, 36 fresh *Amanita* specimens were collected from Yanshan Mountains. There were 20 herbarium specimens were loaned from the Herbarium Mycologicum Academiae Sinicae (HMAS, Institute of Microbiology, Chinese Academy of Sciences) for further research. On the basis of morphological examination and inference of phylogeny, three new species and nine known species were reported herein. The study aimed to determine the taxonomic status and phylogenetic position of *Amanita* species represented by these specimens so as to establish a comprehensive database of macrofungal diversity in Yanshan Mountains, especially *Amanita* species, and to use this database as a basis for future studies on macrofungal systematics, fungal toxin, diversity, and ecology in this region.

## 2 Materials and methods

### 2.1 Sample collection and morphological analyses

The specimen collection area is shown in **Figure 1** (Figure and data provided by the Chinese Academy of Environmental Sciences). The specimens were collected during 2019–2022 from Yanshan Mountains and photographed in the field. Macroscopic features of fresh specimens such as colors and odors were noted. Color codes and designations were assigned after referring to the website ColorHexa (https://www.colourhexa.com). The specimens were dried using a Dorrex dryer at 50°C for approximately 12 h and deposited in the Herbarium of the College of Life Science, Capital Normal University, Beijing, China (BJTC). Other 20 herbarium specimens from the research area were obtained from the HMAS.

To observe microscopic characters, thin sections of the dried material were mounted in 3% KOH or sterilized water. Then, the materials were stained with 1% Congo red to increase the visibility of the structures. Microscopic features (e.g., basidiospores, pileipellis, and volval remnants) were observed and measured under a light microscope (Olympus DP71, Tokyo, Japan). Basidiospore measurements of new species are presented as (a)b − c(d). Among them, b − c, a, and d represent a minimum of 90% of the measured values, minimum extreme values, and maximum extreme values, respectively. Q represents the length/width ratio of the basidiospores, and Q_m_ is the average Q values of all basidiospores measured (Bas, 1969). Q_m_ values ± sample standard deviations are provided. The descriptive terms are in accordance with Cui et al. (2018).

### 2.2 DNA extraction, and PCR amplification and sequencing

DNA was extracted using the M5 Plant Genomic DNA Kit (Mei5 Biotechnology, Co., Ltd., Beijing, China). The extracted DNA was solubilized in 1× TE buffer/sterile water and stored at −20°C for further use. The following primer sets were used for amplification: nrITS1f/nrITS4 (White et al., 1990; Gardes and Bruns, 1993) for the nuclear ribosomal DNA internal transcribed spacer (nrITS rDNA) region, LR5/LR0R (Vilgalys and Hester, 1990) for the large subunit nuclear rDNA (nrLSU rDNA) region, rpb2-6f/rpb2-7r (Cai et al., 2014) for the second largest subunit of the RNA polymerase II (*rpb 2*) region, Am-b-tubulin F/Am-b-tubulin R (Cai et al., 2014) for the beta-tubulin (*β-tubulin*) region, and EF1-983F/EF1-1567R (Rehner and Buckley, 2005) for the translation elongation factor 1-α (*tef1-α*) region. PCRs were performed in a reaction volume of 25 μL. The obtained DNA was subjected to Sanger dideoxy sequencing (Sangon Biotechnology, Co., Ltd, Shanghai, China). The new sequences obtained in this study were deposited in GenBank (https://www.ncbi.nlm.nih.gov). **Table 1** lists the accession numbers of the sequences used for phylogenetic analysis.

**Table 1:** Information of sequences used in the nrITS-nrLSU*-rpb2-tef1-α* phylogenetic analysis in this study.

Taxa	Voucher	Location	GenBank accession numbers			
			nrITSi	nrLSU	*rpb2*	*tef1-α*
*Amanita* cf. *angustilamellata*	HKAS89451	Mount Taishan, Shandong, China	MH508292	MH486431	MH485910	MH508716
*Amanita* cf. *angustilamellata*	HKAS83453	Puer, Yunnan, China	—	MH486430	—	—
*Amanita albidostipes*	HKAS57358(T)	Baoshan, Yunnan, China	MH508500	MH486756	—	—
*Amanita alboradicata*	MHHNU10531 (T)	Jilin, China	MW016759	MW016757	MW546614	MW546620
*Amanita altipes*	HKAS91125	Ganzi, Sichuan, China	MH508254	MH486367	MH485862	MH508669
*Amanita aporema*	FRI62674	Malaysia	KU714575	KU714551	KU714593	KU714538
*Amanita ballerina*	OR1014	Thailand	KY747466	—	KY656883	—
*Amanita ballerina*	OR1026(T)	Thailand	KY747467	—	KY656884	—
*Amanita battarrae*	HKAS92090	Mount Changbai, Jilin, China	MH508266	MH486388	MH485880	MH508689
*Amanita bisporigera*	RET 377-9	Tennessee, USA	KJ466374	KJ466434	—	KJ481936
***Amanita borealis***	**BJTC Z110**	**Changping, Beijing, China**	**OR058499**	**OR042383**	**OR051502**	**OR051531**
***Amanita borealis***	**BJTC L169 (T)**	**Pinggu, Beijing, China**	**OR058500**	**OR042384**	**OR051503**	**OR051532**
*Amanita breckonii*	NY00066695 (T)	USA	—	KJ535440	—	—
*Amanita brunneitoxicaria*	BZ2015-01	Thailand	NR_151655	—	KY656879	—
*Amanita brunneitoxicaria*	BZ2015-02	Thailand	KY747463	—	KY656880.	—
*Amanita* aff. *brunneofuliginea*	MB-000633 (duplicate HKAS84873)	Germany	MH508252	MH486364	MH485861	MH508667
*Amanita brunneofuliginea*	HKAS89226	Daofu, Sichuan, China	MH508269	MH486393	MH485885	MH508693
***Amanita brunneola***	**BJTC Z087**	**Changping, Beijing, China**	**OR058501**	**OR042385**	**—**	**OR051533**
***Amanita brunneola***	**BJTC C650 (T)**	**Miyun, Beijing, China**	**OR058502**	**OR042386**	**OR051504**	**OR051534**
*Amanita brunneomaculata*	HKAS68393	Lijiang, Yunnan, China	MH508278	MH486410	MH485892	MH508698
*Amanita brunneostrobilipes*	HKAS60291 (T)	Hainan, China	MH508281	MH486415	–	MH508703
*Amanita caesarea*	HKAS96166	Italy	MH508283	MH486418	MH485898	MH508705
*Amanita caesareoides*	HKAS71021	Japan	MH508284	MH486419	MH485899	MH508706
*Amanita caesareoides*	HKAS92009	Benxi, Liaoning, China	MH508285	MH486421	MH485901	MH508708
***Amanita caesareoides***	**BJTC 630**	**Yanqing, Beijing, China**	**OR058503**	**OR042387**	**OR051505**	**OR051535**
***Amanita caesareoides***	**BJTC C654**	**Xinglong, Hebei, China**	**OR058504**	**OR042388**	**OR051506**	**OR051536**
*Amanita caojizong*	HKAS79673 (T)	Yunnan, China	MH508291	MH486429	MH485908	MH508714
*Amanita castanea*	MFLU 15-1424	Thailand	KU904823	KU877539	—	—
*Amanita changtuia*	HKAS92100	Mudanjiang, Heilongjiang, China	MH508299	MH486442	MH485919	MH508724
*Amanita chepangiana*	HKAS56718	Yunnan, China	KU714569	KU714545	KU714588	KU714534
*Amanita chiui*	HKAS76328	Yanyuan, Sichuan, China	MH508303	MH486447	MH485930	MH508727
***Amanita chiui***	**BJTC L130**	**Pinggu, Beijing, China**	**OR058505**	**OR042389**	**—**	**OR051537**
*Amanita cinctipes*	HKAS78465	Guangdong, China	MH508305	MH486449	–	–
*Amanita cinctipes*	HKAS101388	Heishiding, Guangdong, China	MH508304	MH486448	—	—
*Amanita cingulata*	HKAS75600 (T)	Yanling, Zhuzhou, China	MH508310	NG_058602	—	—
*Amanita citrina*	HKAS101397	France	MH508311	MH486456	MH485936	MH508732
*Amanita citrinoannulata*	HKAS83459 (T)	Chongqing, China	MH508318	MH486464	MH485944	MH508740
*Amanita citrinoindusiata*	HKAS100522 (T)	Lijiang, Yunnan, China	MH508320	MH486468	MH485947	MH508744
*Amanita collariata*	MHHNU31095	Hunan, China	OM955206	OM955204	OM949814	OM949813
*Amanita concentrica*	HKAS87061	Yunnan, China	MH508327	KR824785	KR824794	KR824827
*Amanita cruzii*	BARONI8998 (T)	Dominican Republic	KC855222	KC855222	—	MH508750
*Amanita detersa*	HKAS71476 (T)	Lushui, Yunnan, China	MH508328	MH486475	MH485954	MH508752
*Amanita eijii*	HKAS70229	Yunnan, China	MH508333	MH486484	MH485963	MH508761
*Amanita elata*	HKAS83449	Xishuangbanna, Yunnan, China	MH508334	MH486486	MH485965	MH508763
*Amanita elliptica*	HKAS79602	Hainan. China	MH508335	MH486487	–	MH508764
*Amanita elongata*	RET 384-5	Canada	MH508337	MH486489	MH485967	MH508766
*Amanita esculenta*	HKAS89035	Xishuangbanna, Yunnan, China	MH508338	MH486490	MH485968	MH508767
*Amanita exitialis*	HKAS75774	Mount Baiyun, Guangdong, China	JX998027	JX998052	KJ466591	JX998001
*Amanita farinosa*	HKAS67958	Tengchong, Yunnan, China	MH508341	MH486498	MH485973	MH508773
*Amanita flavipes*	HKAS57650	Dali, Yunnan, China	MH508343	MH486501	MH485975	MH508776
*Amanita flavoconia*	RET 485-6	New York, USA	MH508351	MH486514	MH485985	MH508790
*Amanita flavopantherina*	HKAS82613	Shangri-La, Yunnan, China	MH508355	MH486519	MH485989	MH508795
*Amanita fritillaria*	HKAS91952	Xishuangbanna, Yunnan, China	MH508367	MH486548	MH486015	MH508820
*Amanita frostiana*	RET7-25-92-E	USA		AF024453		
*Amanita fuliginea*	HKAS75780	Heishiding, Guangdong, China	JX998023	JX998048	KJ466595	JX997995
*Amanita fuligineoides*	HKAS52727	Hunan, China	JX998024	JX998047	KJ466599	
*Amanita fulva*	HKAS96168	Austria	MH508371	MH486555	MH486022	MH508826
*Amanita fulva*	HKAS96168	Australia	MH508371	MH486555	MH486022	MH508826
*Amanita fulvopyramidalis*	MHHNU10581	Zhejiang, China	–	MW471097	MW546619	MW546625
*Amanita fulvopyramidalis*	MHHNU8814 (T)	Hunan, China	MT878220	MT878535	MW546617	MW546623
*Amanita fuscoflava*	HKAS59800 (T)	Limushan, Hainan, China	MH508372	MH486557	MH486023	MH508827
*Amanita fuscosquamosa*	PDD92862	Australia	MH508373	MH486558	—	MH508828
*Amanita gemmata*	C. Bas8942 L	Unknown		AF024457		
*Amanita griseofarinosa*	HKAS80926	Hubei, China	MH508375	MH486559	MH486025	MH508830
*Amanita griseofolia*	HKAS38159 (T)	Yunnan, China	AY436448	AY436488	—	—
*Amanita griseopantheirna*	HKAS83560 (T)	Basu, Tibet, China	MH508385	MH486573	—	MH508842
*Amanita griseorosea*	HKAS77332 (T)	Mount Limu, Hainan, China	KJ466411	KJ466474	—	KJ481992
*Amanita griseoumbonata*	HKAS92103 (T)	Mudanjiang, Heilongjiang, China	MH508389	MH486578	MH486040	MH508847
*Amanita gymnopus*	HKAS71618	Yunnan, China	MH508393	MH486582	MH486044	MH508851
*Amanita hamadae*	HKAS79076	Heishiding, Guangdong, China	MH508394	MH486584	MH486046	—
*Amanita heishidingensis*	HKAS76122	Guangdong, China	NR_151651	KC429045	–	–
*Amanita heishidingensis*	HKAS81484	Guangdong, China	KJ922999	KJ922993	MH486049	MH508854
*Amanita hemibapha*	HMAS 54784	Ninger, Yunnan, China	—	MH486587	—	—
*Amanita hunanensis*	HKAS100632	Jinzhai, Anhui, China	MH508396	MH486588	MH486050	MH508856
*Amanita ibotengutake*	HKAS56045	Xifeng, Jilin, China		MH486589		MH508857
*Amanita imazekii*	HKAS92011	Benxi, Liaoning, China	MH508398	MH486592	MH486053	MH508860
*Amanita incarnatifolia*	HKAS100593	Anhui, China	MH508400	MH486594	MH486056	MH508862
*Amanita japonica*	TMI26147	Japan	KJ922994	KJ922990	–	–
*Amanita javanica*	FRIM:61503	Malaysia	KU714572	KU714548	—	KU714536
*Amanita kitamagotake*	EN-4	Japan	AB721450	AB721450	—	—
*Amanita kotohiraensis*	HKAS100577	Anhui, China	MH508415	MH486614	MH486068	MH508874
*Amanita lippiae*	RET418-2	Brazil	NR_154692	KP258992	—	—
*Amanita liquii*	HKAS36611	Yunnan, China	AY436462	AY436493	—	—
*Amanita longistriata*	HKAS68331	Lanping, Yunnan, China	MH508428	MH486631	MH486081	MH508888
*Amanita longistriata*	HKAS54578	Lijiang, Yunnan, China	—	MH486629	MH486079	—
*Amanita melleialba*	HKAS83446 (T)	Puer, Yunnan, China	MH508430	KR824767	KR824792	KR824813
*Amanita melleiceps*	HKAS80145	Macheng, Hubei, China	MH508432	MH486640	MH486089	MH508897
*Amanita minutisquama*	HKAS100504 (T)	Hainan, China	MH508435	MH486644	MH486095	MH508902
*Amanita mira*	HKAS91953	Mengla, Yunnan, China	MH508437	MH486646	MH486097	—
*Amanita molliuscula*	HKAS75555	Shennongjia, Hubei, China	KJ466408	KJ466471	KJ466638	KJ481973
*Amanita molliuscula*	HKAS77324 (T)	Zhouzhi, Shaanxi, China	KJ466409	KJ466472	KJ466639	KJ481974
*Amanita molliuscula*	HMJAU20469	Mount Changbai, Jilin, China	KJ466410	KJ466473	KJ466640	KJ481975
*Amanita muscaria*	HKAS61888	Huzhong, Heilongjiang, China	MH508439	MH486651	MH486100	MH508908
*Amanita muscaria*	HMJAU4549	Kyrov, Russia	MH508440	KR824787	—	KR824829
***Amanita muscaria***	**BJTC L491**	**Chicheng, Hebei, China**	**OR058506**	**OR042390**	**—**	**OR051538**
*Amanita neoovoidea*	HKAS89025	Yunnan, China	MH508445	MH486656	MH486106	MH508913
*Amanita oberwinkleriana*	HKAS77330	Hainan, China	KJ466380	KJ466441	KJ466606	KJ481946
***Amanita oberwinkleriana***	**BJTC C659**	**Pinggu, Beijing, China**	**—**	**OR042391**	**OR051507**	**OR051539**
***Amanita oberwinkleriana***	**BJTC Z328**	**Jizhou, Tianjin, China**	**—**	**OR042392**	**—**	**OR051540**
***Amanita oberwinkleriana***	**BJTC Z318**	**Jizhou, Tianjin, China**	**—**	**OR042393**	**OR051508**	**OR051541**
***Amanita oberwinkleriana***	**BJTC Z862**	**Jizhou, Tianjin, China**	**—**	**OR042394**	**—**	**OR051542**
***Amanita oberwinkleriana***	**BJTC C320**	**Xinglong, Hebei, China**	**—**	**OR042395**	**—**	**OR051543**
***Amanita oberwinkleriana***	**HMAS 263406**	**Yanqing, Beijing, China**	**OR058507**	**—**	**—**	**—**
***Amanita oberwinkleriana***	**HMAS 253800**	**Mentougou, Beijing, China**	**OR058508**	**OR042396**	**OR051509**	**OR051544**
***Amanita oberwinkleriana***	**HMAS 253801**	**Mentougou, Beijing, China**	**—**	**OR042397**	**OR051510**	**OR051545**
***Amanita oberwinkleriana***	**HMAS 253802**	**Mentougou, Beijing, China**	**—**	**—**	**OR051511**	**—**
***Amanita oberwinkleriana***	**HMAS 253796**	**Mentougou, Beijing, China**	**OR058509**	**OR042398**	**OR051512**	**OR051546**
*Amanita ochracea*	HKAS87986	Shangri-La, Yunnan, China	MH508454	MH486686	MH486123	—
*Amanita ocreata*	HKAS79686	California, USA	KJ466381	MH486688	KJ466607	KJ481947
*Amanita olivaceofusca*	HKAS97581 (T)	Lancang, Yunnan, China	MH508457	MH486691	MH486127	—
*Amanita orienticrocea*	HKAS90455 (T)	Jingdong, Yunnan, China	MH508465	MH486701	MH486133	—
*Amanita orientifulva*	HKAS87937	Shangri-La, Yunnan, China	MH508468	MH486704	MH486136	—
*Amanita orientigemmata*	HKAS80978	Lanping, Yunnan, China	MH508469	MH486708	MH486140	
*Amanita orsonii*	HKAS52264	Kunming, Yunnan, China	MH508474	MH486714	MH486145	—
*Amanita ovalispora*	HKAS79625	Heishiding, Guangdong, China	MH508479	MH486722	MH486150	—
*Amanita ovalispora*	HKAS101406	Hainan, China	MH508478	MH486720	MH486148	—
***Amanita ovalispora***	**BJTC Z057**	**Changping, Beijing, China**	**—**	**OR042399**	**OR051513**	**OR051547**
***Amanita ovalispora***	**BJTC Z311**	**Jizhou, Tianjin, China**	**OR058510**	**OR042400**	**OR051514**	**OR051548**
*Amanita pachycolea*	HKAS101422	USA	MH508480	MH486724	MH486152	—
*Amanita pallidocarnea*	HKSA97678	Lancang, Yunnan, China	MH508482	MH486728	MH486156	—
*Amanita pallidorosea*	HKAS82350	Mount Taishan, Shandong, China	MH508485	MH486737	MH486163	—
*Amanita pallidorosea*	HKAS61937	Meixian, Shaanxi, China	KJ466382	KJ466443	KJ466609	KJ481949
*Amanita pallidorosea*	HKAS82350	Shandong, China	MH508485	MH486737	MH486163	MH508971
*Amanita pallidorosea*	HKAS82350	Shandong, China	MH508485	MH486737	MH486163	MH508971
*Amanita pantherina*	MB-102863 (duplicate HKAS84852)	Germany	MH508488	MH486743	MH486167	—
*Amanita parvifritillaria*	HKAS83737 (T)	Ailaoshan, Yunnan, China	MH508494	MH486749	MH486173	—
*Amanita parvipantherina*	HKAS67828	Yunnan, China	MH508496	MH486750	MH486174	MH508979
*Amanita persicina*	RET151-4	USA	NR_154668	EU071969		EU071862
*Amanita phalloides*	HKAS75773	California, USA	JX998031	JX998060	KJ466612	JX998000
*Amanita porphyria*	HKAS92088	Changbai Mountain, Jilin, China	MH508506	MH486761	MH486180	—
*Amanita* aff. *princeps*	TRTC-150309	Thailand	JX844734	KF877274	—	KF877160
*Amanita princeps*	FRI:62849	Malaysia	KU714576	KU714552	KU714594	KU714539
*Amanita pseudopantheirna*	HKAS80007 (T)	Binchuan, Yunnan, China	MH508514	MH486777	MH486191	—
*Amanita pseudoporphyria*	HKAS89074	Yunnan, China	MH508524	MH486785	MH486199	MH509012
*Amanita pseudoprinceps*	HKAS97523 (T)	Heishiding, Guangdong, China	MH508527	MH486788	MH486202	—
*Amanita pseudosychnopyramis*	HKAS87999 (T)	Lijiang, Yunnan, China	MH508530	KR824778	KR824790	KR824824
*Amanita pseudovaginata*	HKAS70138	Mount Wuliang, Yunnan, China	MH508531	MH486791	MH486205	—
*Amanita pyramidata*	HKAS87943 (T)	Yunnan, China	MH508535	MH486795	MH486209	MH509021
*Amanita regalis*	HKAS56699	Czech Republic	MH508537	MH486797		
*Amanita retenta*	HKAS70020 (T)	Lijiang, Yunnan, China	MH508543	MH486802	MH486215	—
*Amanita rimosa*	HKAS75777	Baisha, Hainan, China	JX998018	JX998044	KJ466615	JX998005
*Amanita roseitincta*	RET 284-10	Texas, USA	MH508550	KC855224	—	—
*Amanita roseolifolia*	HKAS101403 (T)	Hainan, China	MH508548	MH486807	MH486219	MH509032
*Amanita rubescens*	HKAS100631	Anhui, China	MH508554	MH486810	–	MH509037
*Amanita rubescens*	HKAS92034	Anshan, Liaoning, China	MH508558	MH486815	MH486226	—
*Amanita rubiginosa*	HKAS52216 (T)	Yunnan, China	MH508561	MH486817	MH486229	MH509045
*Amanita rubroflava*	HKAS83089 (T)	Lingbaoshan, Yunnan, China	MH508568	MH486827	MH486238	—
*Amanita rubrovolvata*	BZ2015-68	Thailand	KY747465	KY747477	KY656882	
*Amanita rufoferruginea*	HKAS79616	Guangdong, China	MH508579	MH486842	MH486252	MH509068
*Amanita sepiacea*	HKAS56799	Baoshan, Yunnan, China	MH508584	MH486847	MH486256	—
*Amanita shennongjiana*	HKAS75553(T)	Shennongjia, Hubei, China	MH508590	MH486862	MH486270	—
*Amanita siamensis*	HKAS83680	Lingbaoshan, Yunnan, China	—	MH486865	MH486272	—
*Amanita similis*	FRI:3740	Malaysia	KU714566	JF710796	—	KU714531
*Amanita sinensis*	HKAS74388	Xundian, Kunming, Yunnan	MH508596	MH486870	MH486277	—
*Amanita sinocitrina*	HKAS100530	Suiyang, Guizhou, China	MH508598	MH486873	MH486279	—
*Amanita* sp.	HKAS101390	Heishiding, Guangdong, China	MH508603	MH486878	MH486284	MH509100
***Amanita* sp.**	**BJTC S233**	**Yanqing, Beijing, China**	**OR058511**	**OR042401**	**OR051515**	**OR051549**
***Amanita* sp.**	**HMAS 26491**	**Mentougou, Beijing, China**	**OR058512**	**OR042402**	**—**	**OR051550**
*Amanita spissa*	HKAS92089	Changbai mountain, Jilin, China	MH508612	MH486893	MH486296	MH509115
*Amanita spissacea*	HKAS57649	Dali, Yunnan, China	KJ466373	KJ466480	KJ466645	KJ481980
*Amanita spissacea*	HKAS57754	Yulong, Yunnan, China	MH508609	MH486886	MH486290	MH509108
*Amanita squarrosipes*	HKAS76359 (T)	Muli, Sichuan, China	MH508613	MH486894	MH486297	MH509116
*Amanita suballiacea*	RET 490-1	Connecticut, USA	KJ466420	KJ466485	KJ466601	KJ481941
*Amanita subfrostiana*	HKAS58750	Sichuan, China	–	MH486897	MH486299	MH509119
*Amanita subfrostiana*	HKAS57042	Yunnan, China	JN943173	JN941162	JQ031118	KJ482003
*Amanita subfuliginea*	HKAS77326	Lechang, Guangdong, China	KJ466404	KJ466467	KJ466636	KJ481971
***Amanita subglobosa***	**HMAS 253798**	**Miyun, Beijing, China**	**OR058513**	**OR042403**	**OR051516**	**OR051551**
*Amanita subglobosa*	HKAS67914	Baoshan, Yunnan, China	MH508619	MH486902	MH486303	MH509123
*Amanita subjunquillea*	HKAS75770	Meixian, Shaanxi, China	JX998034	JX998062	KJ466653	JX997999
*Amanita subjunquillea*	HKAS75771	Shennongjia, Hubei, China	JX998032	JX998063	KJ466654	JX997997
***Amanita subjunquillea***	**BJTC 217**	**Yanqing, Beijing, China**	**OR058514**	**OR042404**	**OR051517**	**OR051552**
***Amanita subjunquillea***	**BJTC 085**	**Yanqing, Beijing, China**	**OR058515**	**OR042405**	**—**	**—**
***Amanita subjunquillea***	**BJTC C558**	**Huairou, Beijing, China**	**OR058516**	**OR042406**	**OR051518**	**OR051553**
***Amanita subjunquillea***	**BJTC 033**	**Yanqing, Beijing, China**	**OR058517**	**OR042407**	**OR051519**	**OR051554**
***Amanita subjunquillea***	**BJTC 704**	**Yanqing, Beijing, China**	**OR058518**	**OR042408**	**—**	**—**
***Amanita subjunquillea***	**BJTC Z276**	**Xinglong, Hebei, China**	**OR058519**	**OR042409**	**OR051520**	**OR051555**
***Amanita subjunquillea***	**BJTC 112**	**Yanqing, Beijing, China**	**OR058520**	**OR042410**	**—**	**OR051556**
***Amanita subjunquillea***	**BJTC Z172**	**Shunyi, Beijing, China**	**OR058521**	**OR042411**	**OR051521**	**OR051557**
***Amanita subjunquillea***	**HMAS 253775**	**Mentougou, Beijing, China**	**OR058522**	**OR042412**	**OR051522**	**OR051558**
*Amanita submembranacea*	MB-001174 (duplicate HKAS84857)	Germany	MH508626	MH486916	—	MH509135
*Amanita subpallidorosea*	HKAS77350	Taichung, Taiwan, China	KJ466400	KJ466462	KJ466631	KJ481966
*Amanita subpallidorosea*	LHJ140923-41 (T)	Guizhou, China	KP691683	KP691692	KP691701	KP691670
*Amanita subparcivolvata*	MHHNU32907 (T)	Hunan, China	OM955207	OM955205	OM949819	—
*Amanita subparcivolvata*	MHHNU33169	Hunan, China	OM955215	OM955217	OM949820	—
*Amanita subparvipantherina*	HKAS56986 (T)	Yongping, Yunnan, China		KR824776		KR824820
*Amanita sychnopyramis* f. *subannulata*	HKAS26144	Yunnan, China	—	AF024480	—	—
*Amanita sychnopyramis* f. *sychnopyramis*	HKAS83454	Wenshan, Yunnan, China	—	MH486927	—	MH509144
*Amanita timida*	HKAS83228	Guangdong, China	MH508636	MH486930	MH486323	MH509147
*Amanita torrendii*	LOU Fungi 17408	Spain	GQ925386	GQ925369	—	—
*Amanita umbrinolutea*	HKAS89201	Luhuo, Sichuan, China	MH508637	MH486933	MH486326	MH509150
***Amanita vaginata* var. *vaginata***	**HMAS 253281**	**Mentougou, Beijing, China**	**OR058523**	**OR042413**	**OR051523**	**—**
***Amanita vaginata* var. *vaginata***	**BJTC Z521**	**Huairou, Beijing, China**	**OR058524**	**OR042414**	**—**	**—**
***Amanita vaginata* var. *vaginata***	**BJTC 682**	**Yanqing, Beijing, China**	**OR058525**	**OR042415**	**—**	**—**
***Amanita vaginata* var. *vaginata***	**BJTC 677**	**Yanqing, Beijing, China**	**OR058526**	**OR042416**	**OR051524**	**—**
*Amanita vaginata* var. *vaginata*	H.A.v.d.Aa s.n	Unknow	—	AF024482	—	—
*Amanita velatipes*	RET 489-2	Michigan, USA	MH508643	MH486938	MH486331	MH509155
*Amanita vestita*	HKAS79687	Hainan, China	MH508647	KJ466494	KJ466662	KJ481995
*Amanita virgineoides*	HKAS100518	Henan, China	MH508648	MH486944	MH486339	MH509165
*Amanita virosa*	HKAS56694	Juva, Finland	JX998030	JX998058	KJ466664	JX998007
*Amanita* cf. *xanthogala*	HKAS84707	Yunnan, China	MH508298	MH486441	MH485918	MH508723
***Amanita yanshanensis***	**BJTC Z083**	**Changping, Beijing, China**	**OR058527**	**OR042417**	**OR051525**	**OR051559**
***Amanita yanshanensis***	**BJTC Z760**	**Changping, Beijing, China**	**OR058528**	**OR042418**	**OR051526**	**OR051560**
***Amanita yanshanensis***	**BJTC Z049 (T)**	**Changping, Beijing, China**	**OR058529**	**OR042419**	**OR051527**	**OR051561**
***Amanita yanshanensis***	**BJTC Z824**	**Changping, Beijing, China**	**OR058530**	**OR042420**	**—**	**OR051562**
***Amanita yanshanensis***	**BJTC C182**	**Pinggu, Beijing, China**	**OR058531**	**OR042421**	**—**	**OR051563**
***Amanita yanshanensis***	**BJTC Z815**	**Changping, Beijing, China**	**OR058532**	**OR042422**	**OR051528**	**OR051564**
***Amanita yanshanensis***	**BJTC Z819**	**Changping, Beijing, China**	**OR058533**	**OR042423**	**OR051529**	**OR051565**
***Amanita yanshanensis***	**BJTC Z820**	**Changping, Beijing, China**	**OR058534**	**OR042424**	**OR051530**	**OR051566**
*Amanita yenii*	HKAS87047	Yunnan, China	MH508652	MH486951	MH486344	MH509171
*Amanita yuaniana*	HKAS58807	Lijiang, Yunnan, China	MH508653	MH486954	MH486347	MH509174
*Limacella glioderma*	HKAS90169	Heilongjiang, China	MH508658	KT833808	KT833823	KT833836
*Limacellopsis Asiatica*	HKAS76497	Gansu, China	—	KT833811	KT833826	KT833839
*Limacellopsis Asiatica*	HKAS82561 (T)	Aba, Sichuan, China	—	KT833812	KT833827	KT833840
*Myxoderma ochraceoluteum*	MEL2305332	Australia	MH508660	MH486965	MH486358	MH509185
*Myxoderma ochraceoluteum*	MEL2341329	Australia	MH508661	MH486966	MH486359	MH509186

The new generated sequences are emphasized in bold; “–” show no sequence.

The new species is in bold. “—” means no sequence.

### 2.3 Molecular data analyses and species delimitation

The nrITS-nrLSU*-rpb2-tef1-α* multi-locus dataset included 216 ingroup samples. These samples were used to infer the phylogenetic status of our *Amanita* specimens at the phylloclade level. The nrLSU dataset included 204 ingroup samples. These samples were used to analyze the subsection in which the species were located. Furthermore, nrITS was used to infer phylogenetic relationships between new and known *Amanita* species, as GenBank contains a large amount of nrITS sequence data for this genus. These nrITS sequences of new species were divided into different datasets, given that these sequences are too variable to obtain reliable genus-wide comparisons. Based on previous study findings and the GenBank database of the National Center for Biotechnology Information, reference sequences of all *Amanita* species in the dataset were selected for phylogenetic analysis (Cui et al., 2018; Su et al., 2022, **Table 1**). *Limacella glioderma* (Fr.) Maire (HKAS 90169), *Limacellopsis asiatica* Zhu L. Yang, Q. Cai & Y.Y. Cui (HKAS 76497 and 82561), *Myxoderma ochraceoluteum* (P.D. Orton) Zhu L. Yang, Q. Cai & Y.Y. Cui (MEL 2305332), and *M. ochraceoluteum* (MEL2341329) as outgroup refers to Cui et al. (2018).

All sequences were compared using MAFFT v.6 (Katoh and Toh, 2010) and trimmed automatically using Gblocks 0.91b (http://phylogeny.lirmm.fr/phylo_cgi/one_task.cgi?task_type=gblocks) (Dereeper et al., 2008). Bayesian inference (BI) analysis was performed using MrBayes v.3.1.2 (Ronquist and Huelsenbeck, 2003), and maximum likelihood (MI) analysis gene trees were estimated using RAxML 7.4.2 Black Box (Stamatakis, 2006; Stamatakis et al., 2008).

The BI analysis was performed using a Markov chain Monte Carlo (MCMC) algorithm (Rannala and Yang, 1996) and MrBayes 3.1.2 (Ronquist and Huelsenbeck, 2003) based on the best substitution model determined by MrModeltest 2.3 (Nylander, 2004), GTR + I + G for nrITS, nrLSU, *rpb2*, and *tef-1α*. Two MCMC chains were run from random trees for 10,000,000 generations, stopping when the average standard deviations of split frequencies were less than 0.01. Trees were stored for each 1,000 generations. The first 25% of the trees were excluded as the burn-in stage for each analysis. Branches with significant Bayesian posterior probability (BPP) values were then estimated in the resulting trees (Posada and Crandall, 1998). The ML analysis was performed using a GTR + GAMMA + I locus replacement model (Guindon et al., 2010). The branch support was obtained using the bootstrapping (BS) method of 1,000 replications (Hillis and Bull, 1993). Branches with a bootstrap (BS) support of ≥50% and BPP of ≥0.95 were considered significant (Hillis and Bull, 1993).

## 3 Results

### 3.1 Phylogenetic analyses

**Figure 2:**
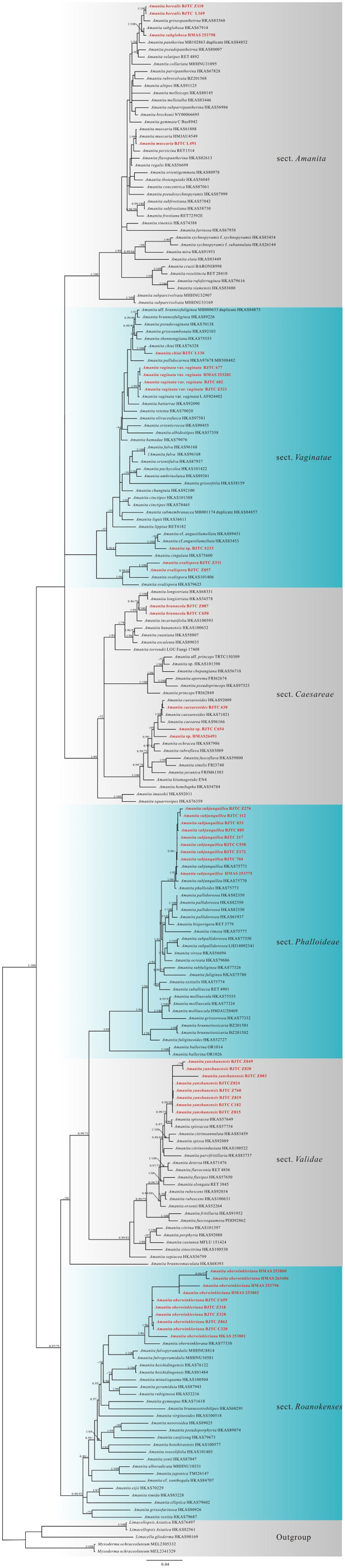
The nrITS-nrLSU*-rpb2-tef1-α* multi-locus phylogenetic tree obtained from the Bayesian analysis.

The nrLSU dataset contained 209 sequences, including 42 newly obtained sequences. The length of the aligned dataset was 790 bp long. The nrLSU phylogenetic analysis results revealed that our specimens belonged to six sections under *Amanita*, namely, sections *Amanita* Pers., *Caesareae* Singer, *Roanokenses* Singer, *Phalloideae* Quél., *Validae* Quél, and *Vaginatae* Quél (**Figure S1**), and were divided into 12 clades. Subsequently, the nrITS-nrLSU*-rpb2-tef1-α* multi-locus phylogenetic analysis was performed to infer the phylogenetic status of our *Amanita* specimens at the phylloclade level (**Figure 2**). The combined nrITS-nrLSU*-rpb2-tef1-α* dataset had 726 sequences, including 143 newly obtained sequences in this study. The aligned dataset was 2,150 bp long including alignment gaps (245 bp for nrITS, 790 bp for nrLSU, 660 bp for *rpb2*, and 365 bp for *tef1-α*). Results of the nrITS-nrLSU*-rpb2-tef1-α* and nrLSU phylogenetic analyses revealed that the subgenera and sections proposed by Cui et al. (2018) were strongly supported with significant BPP values and ML bootstrap (MLB).

The nrITS-nrLSU*-rpb2-tef1-α* multi-locus phylogenetic analysis showed that our specimens were clustered into 14 clades. Moreover, they formed three distinct and strongly supported new branches, which were nested in sections *Amanita*, *Caesareae*, and *Validae*, respectively. These three new lineages were as follows: two specimens (BJTC Z110 and BJTC L169) formed one clade (BPP = 1.00, MLB = 100%) and were closely related to *A. griseopantherina* Yang-Yang Cui, Qing Cai & Zhu L. Yang, *A. pantherina* (DC.) Krombh., and *A. subglobosa* Zhu L. Yang on **Figure 2**. The two specimens (BJTC Z087 and BJTC C650) clustered into a branch with a high support (BPP = 1.00, MLB = 100%), which further clustered into a clade containing *A. longistriata* S. Imai., with moderate support. Eight specimens (BJTC Z049, BJTC Z820, BJTC Z083, BJTC Z824, BJTC Z760, BJTC Z819, BJTC C182, and BJTC Z815) were clustered together, forming a completely supported clade (BPP = 0.99, MLB = 99%). The new branches clustered with *A. spissacea* S. Imai and formed a sister clade in the phylogenetic tree. The nrLSU phylogenetic analysis revealed topologies similar to those of the multi-locus phylogenetic tree, and the specimens also formed three new lineages. Therefore, based on the results of phylogenetic and morphological analyses, these new clades were identified as three new species herein.

In addition, through morphological and phylogenetic analyses, we identified nine known *Amanita* species collected from Yanshan Mountains, northern China, including *A. caesareoides* Lj. N. Vassiljeva, *A. chiui* Yang-Yang Cui, Qing Cai & Zhu L. Yang, *A. muscaria* (L.: Fr.) Lam., *A. oberwinkleriana* Zhu L. Yang & Yoshim. Doi, *A. ovalispora* Boedijn, *A. virosa* Bertillon, *A. subglobosa* Zhu L. Yang, *A. subjunquillea* S. Imai, and *A. vaginata* var. *vaginata* (Bull.) Lam. Because we could not obtain the sequence information of the herbarium specimens (HMAS 40501 and HMAS 40503) of *A. virosa*, we could only identify them through morphological observation.

Although the specimen BJTC S233 was clustered with *A.* cf. *angustilamellata* (HKAS 89451 and HKAS 83453) in the nrITS-nrLSU*-rpb2-tef1-α* and nrLSU phylogenetic analyses, additional specimens are required to elucidate its phylogenetic position and morphological characters. The specimens BJTC C654 and HMAS 26491 probably represented undescribed species. However, they could not be described in the present study because of the poor condition of their basidiomata, inadequate number of specimens, and uncertain phylogenetic position in the nrITS-nrLSU*-rpb2-tef1-α* phylogenetic analysis. Therefore, we only temporarily termed them as *Amanita* sp.

The numbers above the branches represent strong support (BPP ≥0.95 and/or MLB ≥50%). Red font represents t the location of the newly acquired sequences. **Table 1** presents the accession numbers of sequence information used.

### 3.2 Taxonomy

Based on our phylogenetic and morphological data, three new species and nine known species of *Amanita* from Yanshan Mountains are described below.

#### 3.2.1 *Amanita borealis* H. Zhou & C. L. Hou, sp. nov.

**Figure 3:**
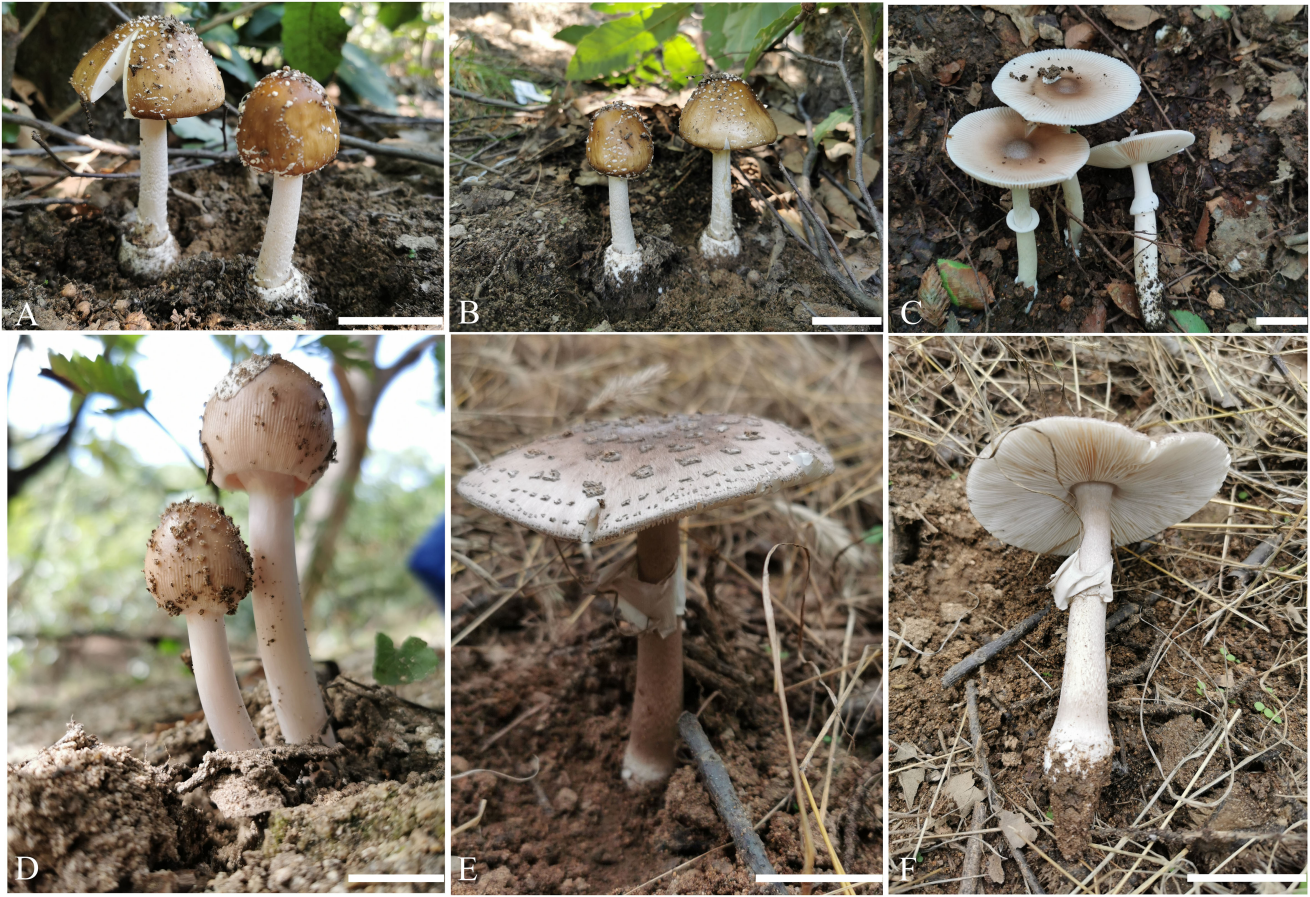
Fresh basidiomata of new species of *Amanita* in this study. **(A, B)** *Amanita borealis* sp. nov. (type, BJTC L169); **(C)** *Amanita brunneola* sp. nov. (type, BJTC C650); **(D)** *Amanita brunneola* sp. nov. (BJTC Z087); **(E, F)** *Amanita yanshanensis* sp. nov. (type, BJTC Z049). Bars: **(A–E)** = 2 cm.

**Figure 4:**
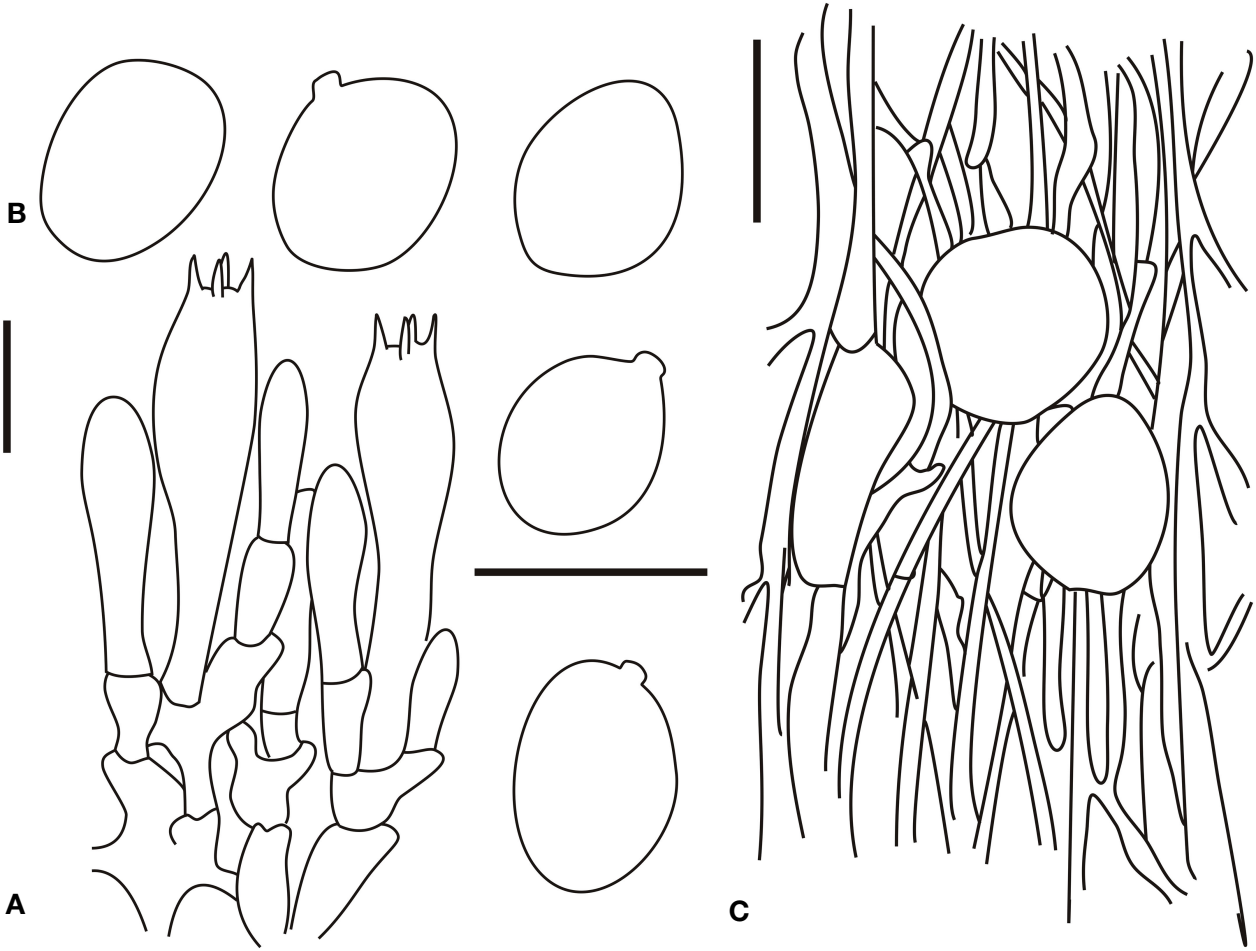
Microscopic characters of *Amanita borealis* (type, BJTC L169). **(A)**. Hymenium and subhymenium; **(B)**. Basidiospores; **(C)**. Longitudinal section of volval remnants on pileus. Bars: **(A, B)** = 10 μm, **(C)** = 40 μm.

**Figures 3A, B**, **4**

**MycoBank: MB 847659**

**Etymology:** The specific epithet “*borealis*” means “northern,” referring to the native occurrence of this species in the Northern China.

**Type:** CHINA. Beijing, Pinggu district, Dongniujiaoyu village, 40.323599 N, 117.156964 E, alt. 477 m, 19 Aug., 2020, coll. G.Q.C., C.L.H. and Y.T.Z. (L169/BJTC L169).

Basidiomata small- to medium-sized. Pileus 2–6 cm in diam., plano-convex to applanate, lacking an obvious depression or umbo at the center; surface brownish (#915b25), brown (#7d4e20) to dark brown (#402810), darker at the center, pyramidal, subverrucose to subconical, dirty white (#f2f2f2) to white (#ffffff) volval remnants on the pileus (ca. 2–8-mm diam.), densely arranged over the disk; margin slightly striate (ca. 0.05–0.1 R), non-appendiculate; trama white (#ffffff), unchanging. Free, crowded, white (#ffffff) lamellae; truncated, plentiful lamellulae. Stipe 5–10 cm long, 0.4–1.1 cm wide at the apex, subcylindric or slightly attenuate upward, surface white (#ffffff), with silk luster, glabrous or covered with concolorous, floccose squamules; white (#ffffff) to yellowish (#ffffe7) context; basal bulb subglobose to fusiform (1.5–2.5-cm diam.), white (#ffffff) to yellowish (#ffffe7); floccose volval remnants on the stipe base, arranged in belts on the lower part of the stipe, and often forms a collar-like or shortly limbate volva on limit between the stipe and basal bulb, white (#ffffff) to yellowish (#ffffe7). Annulus apical, subapical to fugacious, thick, with white (#ffffff) and silk luster on the upper surface. Spore print not observed. Odor indistinct.

Bilateral lamellar trama, 20–60-μm-wide mediostratum, composed of abundant subfusiform, ellipsoid to clavate inflated cells (20–80 × 12–50 μm); abundant, 2–9-μm-wide filamentous hyphae; scarce vascular hyphae. Lateral stratum is composed of abundant subfusiform to ellipsoid inflated cells (20–45 × 6–25 μm), diverging at an angle of ca. 30°–45° to the mediostratum; abundant, 3–8-μm-wide filamentous hyphae. A 30–60-μm-thick subhymenium, with two to three layers of ovoid, subglobose, fusiform, ellipsoid, or irregular cells (10–30 × 8–20 μm). Basidia (30–50 × 7.5–14 μm), slenderly clavate, four-spored, with clamps, hyaline; 3–5-μm-long sterigmata; basidiospores [60/2/2] (8.0–)8.7–9.6(–11.5) × (6.0–)6.5–8.2(–8.5) μm, Q = (1.10–)1.22–1.45(–1.51), Qm = 1.31 ± 0.10, broadly ellipsoid to ellipsoid, thin-walled, hyaline, pale yellow, smooth, small apiculus, inamyloid; sterile lamellar edge, composed of subglobose to ellipsoid or sphaeropedunculate inflated cells (15–40 × 10–30 μm), single and terminal or in chains of 2–3, thin-walled, hyaline; abundant, 3–9-μm-wide filamentous hyphae, irregularly arranged or ± running parallel to the lamellar edge. Pileipellis 50–200 μm thick, gelatinized upper layer (30–75 μm thick), composed of radially, thin-walled, colorless, 2–8-μm-wide filamentous hyphae; lower layer (40–100 μm thick) composed of radially and compactly arranged, colorless, 2–8-μm-wide filamentous hyphae; scarce vascular hyphae. Volval remnants on the pileus are composed of somewhat vertically to irregularly arranged elements: 3–7-μm-wide scarce to scattered, subcolorless, thin-walled, branching, anastomosing filamentous hyphae; globose, subglobose, fusiform to ellipsoid, sometimes irregular, inflated cells (30–70 × 10–55 μm) that are nearly colorless, slightly thick-walled, and terminal or in chains of 2–3; scarce vascular hyphae. Volval remnants on the stipe base similar to that on the pileus, but with more abundant filamentous hyphae and fairly abundant vascular hyphae. Longitudinally acrophysalidic stipe trama; acrophysalides (50–300 × 15–50 μm); scattered to abundant, 3–15-μm-wide filamentous hyphae. Annulus is composed of radially arranged elements: abundant, subglobose, fusiform to ellipsoid inflated cells (20–40 × 10–30 μm) that were hyaline and thin-walled; abundant, 2–7-μm-wide filamentous hyphae that were hyaline and thin-walled. Clamps present in all parts of basidioma.

**Habitat and distribution:** This species is scattered in the broad-leaved forests of *Q. mongolica* Fisch. Ex Ledeb. Basidioma occurs in summer and autumn.

**Additional specimens examined:** CHINA. Beijing, Changping district, Beitaizi, 40.272906 N, 116.420298 E, alt. 149 m, 15 Aug., 2019, coll. H.Z., X.Y.S. and Y.T.Z. (ZH110/BJTC Z110).

**Commentary:** *A. borealis* is well circumscribed by its brownish to dark-brown pileus densely covered with pyramidal, subverrucose to subconical, floccose volval remnants on the stipe base arranged in incomplete belts, and broadly ellipsoid to ellipsoid basidiospores (8.0–11.5 × 6.0–8.5 μm). Furthermore, it is found in association with Fagaceae (*Quercus mongolica*) trees.

*A. borealis* belongs to section *Amanita* and is closely related to *A. griseopantherina*, *A. pantherine*, and *A. subglobosa* on the multi-locus and nrITS phylogenetic trees (**Figures 2**, **S2**). In addition, *A. borealis*, *A. subglobosa*, and *A. pantherine* have similar morphologies. *A. pantherine* is a species originally described from Europe but is not found in China. It has a relatively lower annulus, narrower basidiospores, and no clamps (Cui et al., 2018). From the morphological viewpoint, *A. subglobosa* can be separated from *A. borealis* based on its relatively darker-colored pileus, usually with the presence of clamps and wider basidiospores (8.5–12.0 × 7.0–9.5 μm) (Yang, 1997; Yang, 2005; Yang, 2015; Cui et al., 2018).

#### 3.2.2 *Amanita brunneola* H. Zhou & C. L. Hou, sp. nov.

**Figure 5:**
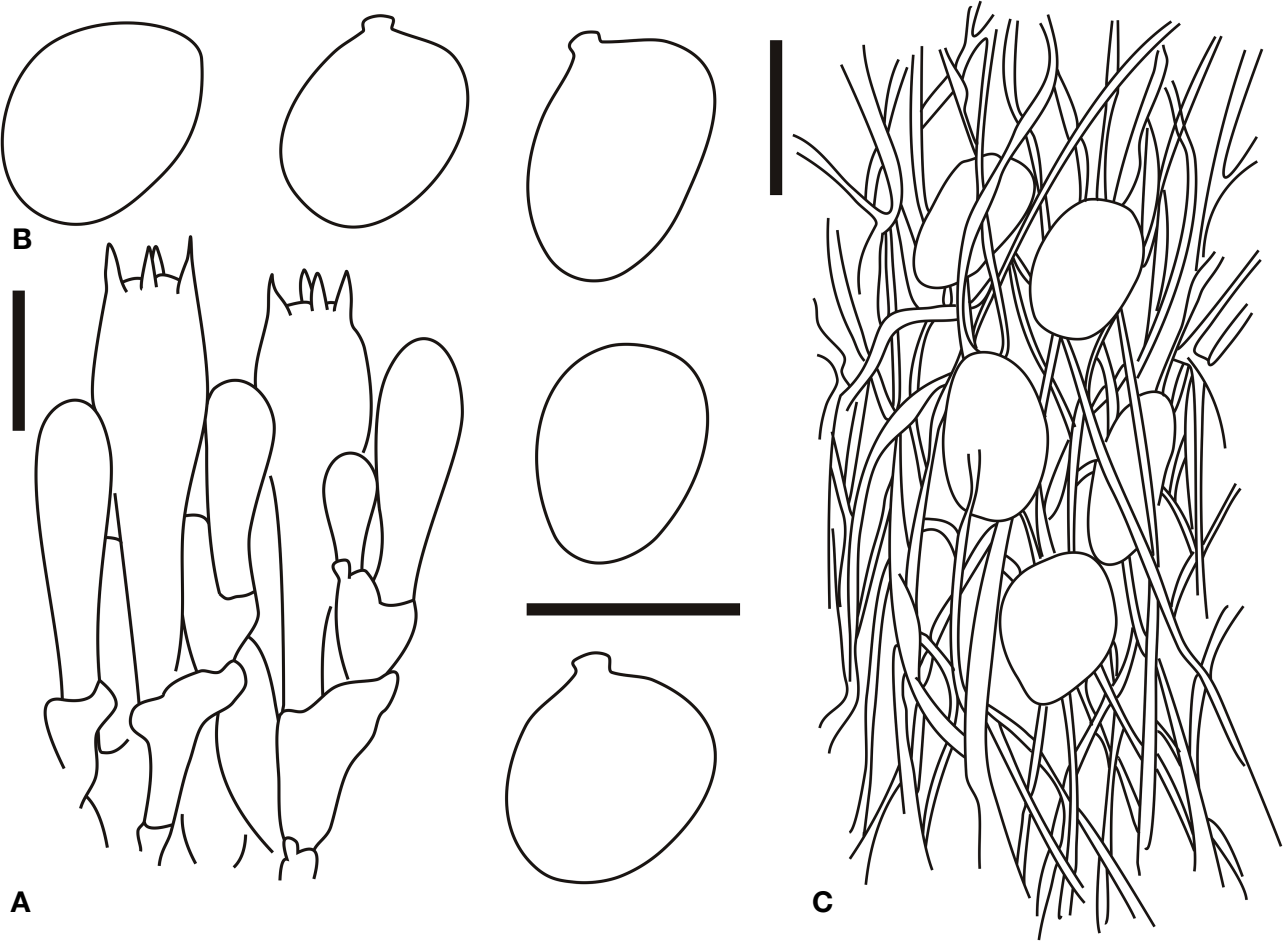
Microscopic characters of *Amanita brunneola* (type, BJTC C650). **(A)**. Hymenium and subhymenium; **(B)**. Basidiospores; **(C)**. Longitudinal section of outer part of volval remnants on stipe base. Bars: **(A, B)** = 10 μm, **(C)** = 40 μm.

**Figures 3C, D**, **5**

**MycoBank: MB 847660**

**Etymology:** The epithet “*brunneola*” refers to the brown tone of pileus.

**Type:** CHINA. Beijing, Miyun district, Heilongtan, 40.560617 N, 116.782229 E, alt. 260 m, 27 Aug., 2020, coll. G.Q.C., C.L.H. and Y.T.Z. (C650/BJTC C650).

Basidiomata small- to medium-sized. Pileus 3–7 cm in diam., convex to applanate, with an umbo at the center, brown tone (#808080) to dark orange (#a5682a) over the disk; verrucose to subconical, dirty white (#f2f2f2) to white (#ffffff) volval remnants on the pileus; margin slightly striate (ca. 0.25–0.3 R), non-appendiculate; trama white (#ffffff), unchanging. Free, crowded, white (#ffffff) to cream (#fffdd0) lamellae; truncated, plentiful lamellulae. Stipe 8–15 cm long, 0.5–1.5 cm wide at the apex, subcylindric or slightly tapering upward, with the apex slightly expanded, white (#ffffff) to pale grayish (#a6a6a6), glabrous above the annulus, and densely covered with gray (#a1a1a1) to dark gray (#888888) squamules under the annulus; white (#ffffff) context, hollow in the center; absence of basal bulb; volva saccate (ca. 2 × 2.5 cm) membranous, both surfaces white (#ffffff) to dirty white (#f2f2f2). Annulus subapical, pendant from attachment ca. 2 cm below the apex of the stipe, membranous, pale grayish upper surface (#808080), brownish gray lower surface (#a5682a). Spore print not observed. Odor indistinct.

Bilateral lamellar trama, 20–60-μm-wide mediostratum, composed of abundant subfusiform, ellipsoid to clavate inflated cells (35–80 × 10–40 μm); abundant, 4–12-μm-wide filamentous hyphae; scarce vascular hyphae. Lateral stratum is composed of abundant subfusiform to ellipsoid inflated cells (25–55 × 9–15 μm), diverging at an angle of ca. 30–45° to the mediostratum; abundant, 2–6-μm-wide filamentous hyphae. A 30–55-μm-thick subhymenium, with two to three layers of subglobose to ellipsoid or irregular cells (10–20 × 8–20 μm). Basidia (**Figure 5A**) 35–50 × 8.5–15 μm, slenderly clavate, four-spored, with clamps, hyaline; 2–5-μm-long sterigmata; basidiospores [60/2/2] (9.5–)9.9–11(–12) × (7.5–)8.5–9.2(–9.5) μm, Q = (1.12–)1.22–1.42(–1.53), Qm = 1.34 ± 0.12, mostly ellipsoid, occasionally broadly ellipsoid, thin-walled, hyaline, smooth, occasionally with a small apiculus, inamyloid; sterile lamellar edge, composed of subglobose to ellipsoid or sphaeropedunculate inflated cells (10–40 × 10–30 μm), single and terminal or in chains of 2–3, thin-walled, hyaline; abundant, 2–5-μm-wide filamentous hyphae, irregularly arranged or ± running parallel to the lamellar edge. Pileipellis 80–150 μm thick, slightly gelatinized upper layer (20–50-μm thick), composed of radially arranged, thin-walled, colorless, 2–5-μm-wide filamentous hyphae; lower layer (40–90-μm thick) composed of radially arranged, colorless to brownish (#a53f2a), 2–8-μm-wide filamentous hyphae; scarce vascular hyphae. Volval remnants on the pileus are composed of inflated cells up to 20–40 × 15–30 μm and abundant filamentous hyphae, subglobose, ovoid to ellipsoid, clavate or sphaeropedunculate, with hyaline to yellowish (#ffff9a) vacuolar pigments; septa with clamps; inner part of volval remnants on the pileus often with conspicuous vascular hyphae. Volval remnants on the stipe base are similar to those on the pileus, but with more abundant filamentous hyphae and fairly abundant vascular hyphae. Longitudinally acrophysalidic stipe trama; acrophysalides (35–280 × 15–50 μm); scattered to abundant, 3–13-μm-wide filamentous hyphae. Annulus is composed of radially arranged elements: scarce, ellipsoid to cylindrical, colorless, thin-walled inflated cells (30–80 × 10–20 μm); very abundant to dominant, 2–8 μm wide, colorless, thin-walled filamentous hyphae; scarce vascular hyphae. Clamps are present in all parts of basidioma.

**Habitat and distribution:** This species is scattered in the broad-leaved forests of *Castanea mollissima* Blume and *Carpinus turczaninowii* Hance. Basidioma occurs in summer and autumn.

**Additional specimens examined:** CHINA. Beijing, Changping district, Dayangshan Mountains National Forest Park, 40.30799 N, 116.424929 E, alt. 260 m, 14 Aug., 2019, coll. H.Z., X.Y.S.and Y.T.Z. (ZH087/BJTC Z087).

**Commentary:** *A. brunneola* is well circumscribed by its brownish to dark orange pileus, with an umbo at the center, covered with verrucose to subconical, densely covered with gray to dark gray squamules under the annulus, and mostly ellipsoid, occasionally broadly ellipsoid basidiospores (9.5–12 × 7.5–9.5 μm). Furthermore, it is found in association with *Fagaceae* and *Betulaceae* trees.

*A. brunneola* belongs to section *Caesareae* and is closely related to *A. longistriata*, *A. fense* M. Mu & L.P. Tang, and *A. incarnatifolia* Zhu L. Yang on the multi-locus and nrITS phylogenetic trees (**Figures 2**, **S3**). *A. brunneola* can be distinguished from *A. fense* and *A. longistriata* on the basis of its longer striations (0.3–0.5 R) on the pileal margin, white to cream lamellae, and a white stipe. Basidiospores of *A. brunneola* (10.0–13.0 × 8.0–11.0 μm) are longer than those of *A. longistriata*, whereas they are shorter than those of *A. fense* (Imai, 1938; Gilbert, 1940; Gilbert, 1941; Hongo, 1959; Yang and Doi, 1999; Yang, 2005; Yang, 2015; Cui et al., 2018; Wu et al., 2021).

#### 3.2.3 *Amanita caesareoides* Lj. N. Vassiljeva, Notul. syst. Sect. cryptog. Inst. bot. Acad. Sci. U. S. S. R. 6: 199 (1950)

**Figures 6A, B**

**Figure 6:**
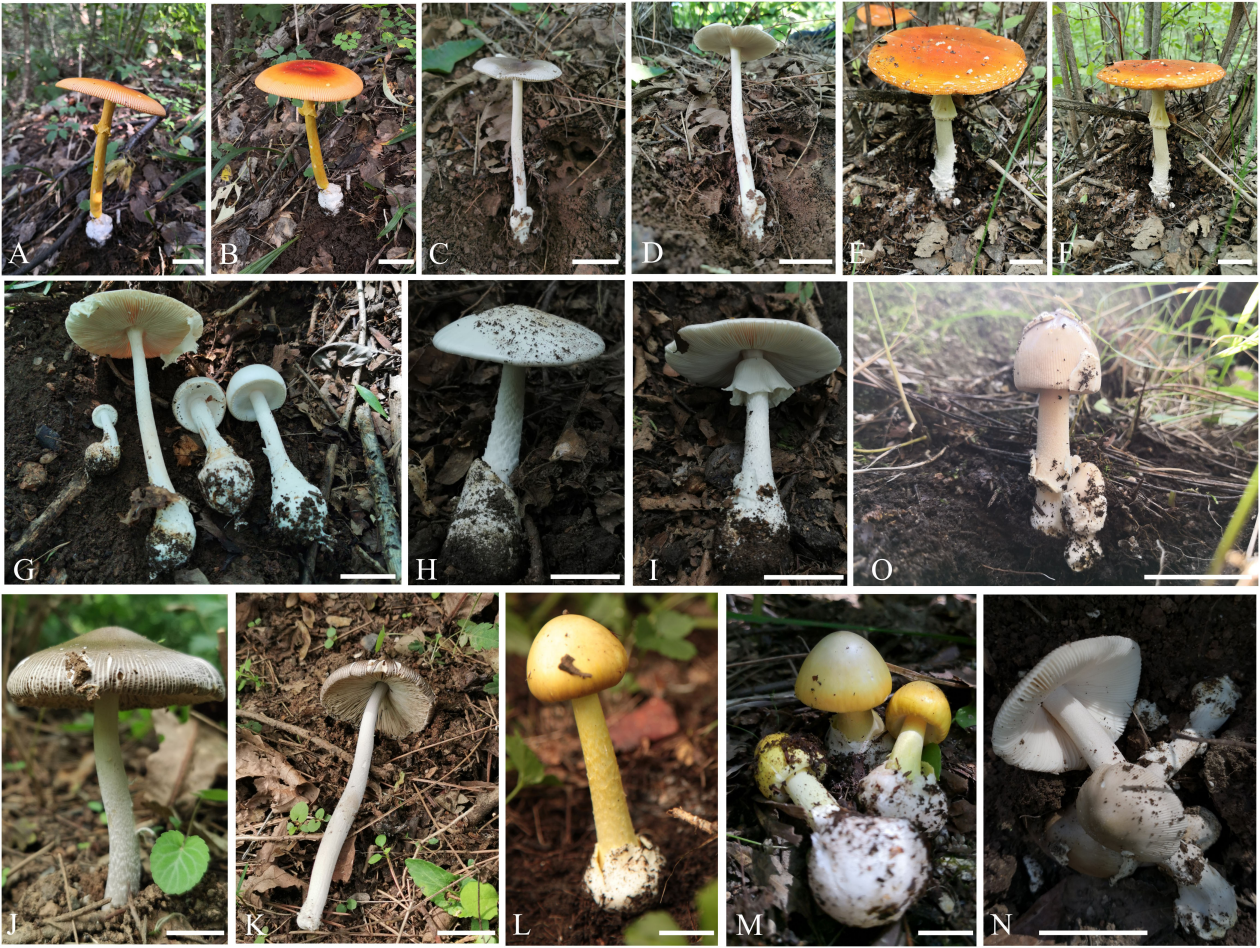
Fresh basidiomata of known species of *Amanita* in this study. **(A, B)** *A caesareoides* (BJTC C630); **(C, D)** *A chiui* (BJTC L130); **(E, F)** *A muscaria* (BJTC L491); **(G)** *oberwinkleriana* (BJTC C320); **(H, I)** *A oberwinkleriana* (BJTC C659); **(J, K)** *A ovalispora* (BJTC Z311); **(L)** *A subjunquillea* (BJTC 085); **(M)** *A subjunquillea* (BJTC 704); **(N)** *A vaginata* var. *vaginata* (BJTC 677); **(O)** *A vaginata* var. *vaginata* (BJTC 682). Bars: **(A–O)** = 2 cm.

Basidiomata small- to medium-sized. Pileus 5–10 cm in diam., applanate, often umbonate at the center, orange-red (#ffa500) to orange (#ffb733); absence of volval remnants on the pileus; margin striate (0.3–0.5 R), non-appendiculate. Cream (#fffdd0) to yellowish (#ffff4d) lamellae; truncated lamellulae. Stipe 8–18 cm long, 0.7–2 cm wide, yellowish (#ffff4d) to orange-red (#ffa500), with its surface covered with snakeskin-shaped, orange (#ffb733) squamules; absence of basal bulb; volval remnants on the stipe base saccate. Annulus subapical, orange-red (#ffa500) to orange (#ffb733). Spore print not observed. Odor indistinct.

Basidia (33–45 × 8–12 μm), clavate, four-spored. Basidiospores [60/4/2] (7.0–)7.5–9.5(–10.5) × (6.0–)6.5–8.2(–8.5) μm, Q = (1.06–)1.12–1.34(–1.40), Qm = 1.23 ± 0.06, broadly ellipsoid, rarely ellipsoid or subglobose, inamyloid. Clamps are present in all parts of basidioma.

**Distribution:** This species is known to be found in northeastern China (Yang, 2015; Cui et al., 2018), India (Bhatt et al., 2003; Bhatt et al., 2017), Japan (Imazeki and Hongo, 1987; Endo et al., 2016), Republic of Korea (Cho et al., 2015), and Russian Far East (Vassiljeva, 1950).

**Habitat and distribution:** It is present individually or is scattered in the broad-leaved forests of *C. viminea* Lindley and *P. davidiana* Dode. Basidioma occurs in summer and autumn.

**Specimens examined:** CHINA. Beijing, Yanqing district, Yudushan Mountains, 40.550099 N, 115.875254 E, alt. 963 m, 20 Aug., 2018, coll. C.L. Hou, H. Zhou and J.Q. Li (630/BJTC 630).

**Commentary:** *A. caesareoides* was first described from the Russian Far East by Vassiljeva (1950). It was subsequently reported from China, India, Japan, and Republic of Korea (Imazeki and Hongo, 1987; Bhatt et al., 2003; Cho et al., 2015; Yang, 2015; Endo et al., 2016; Bhatt et al., 2017). The morphological description of our specimen is consistent with that provided by Vassiljeva (1950). In our multi-locus phylogenetic analysis, the specimen BJTC 630 clustered with *A. caesareoides* (HKAS 92009 and HKAS 71021), forming a completely supported clade (BPP = 1.00, MLB = 88%) (**Figure 2**). Based on these characters, we described our specimen BJTC 630 as *A. caesareoides*. Detailed descriptions, line drawings, and images of *A. caesareoides* can be found in Yang (2015).

#### 3.2.4 *Amanita chiui* Yang-Yang Cui, Qing Cai & Zhu L. Yang, Fungal Diversity 91: 77 (2018)

**Figures 6C, D**

Basidiomata small- to medium-sized. Pileus 3–8 cm in diam., often dark gray (#a9a9a9), brown (#a5682a) to brownish (#d18e4a); volval remnants on the pileus mostly absent or occasionally retained as small, white (#ffffff) patches; margin striate (0.25–0.3 R), non-appendiculate; trama white (#ffffff), unchanging. Free, white (#ffffff) to cream (#fffdd0) lamellae. Stipe 6–10 cm long, 0.5–1.5 cm wide at the apex, white (#ffffff), dirty white (#fcfcfc), brownish (#a5682a) to brown (#cc8236). Annulus absent. Spore print not observed. Odor indistinct.

Basidia (45–65 × 14–17 μm), clavate, four-spored; 4–6-μm-long sterigmata; basal septa lacking clamps. Basidiospores [30/2/1] (9.0–)9.5–12.0(–12.5) × (8.0–)9.0–11.0(–11.5) μm, Q = (1.00–)1.05–1.25(–1.30), Qm = 1.15 ± 0.06, subglobose to broadly ellipsoid, inamyloid, colorless, thin-walled, smooth; small apiculus. Volval remnants on the stipe base are composed of longitudinally arranged elements: abundant to very abundant, branching, anastomosing filamentous hyphae. Clamps are absent in all parts of basidioma.

**Distribution:** This species is known to be found in northwestern and southwestern China (Cui et al., 2018).

**Habitat and distribution:** It is present individually or scattered in the broad-leaved forests of *Castanea mollissima* Blume, with basidioma occurring in summer and autumn.

**Specimens examined:** CHINA. Beijing, Pinggu district, Sizuolou, 40.272187 N, 117.135187 E, elev. 223 m, 16 August 2019, coll. C.L. Hou, G.Q. Cheng and R.T. Zhang (L130/BJTC L130).

**Commentary:** Generally, *A. chiui* is characterized by a dark gray, brown to brownish pileus; a white to dirty white stipe that is often densely covered with brownish squamules; and subglobose to broadly ellipsoid basidiospores (10.0–12.5 × 9.0–11.0 μm) (Cui et al., 2018). Our multi-locus phylogenetic analysis revealed that the specimen BJTC L130 clustered together with *A. chiui* (HKAS 77330, type), forming a completely supported clade (pp = 1.00, MLB = 100%). The nrLSU phylogenetic analysis exhibited topologies similar to those of the multi-locus phylogenetic tree (**Figure S1**). Based on these characters and phylogenetic analysis results, the specimen BJTC L130 was described as *A. chiui*. Detailed descriptions, line drawings, and images of *A. caesareoides* can be found in Cui et al. (2018).

#### 3.2.5 *Amanita muscaria* (L.: Fr.) Lam., Encycl. Méth. Bot. (Paris) 1(1): 111 (1783)

**Figures 6E, F**

Basidiomata small- to large-sized. Pileus 5–15 cm in diam., orange-red (#ffa500); pyramidal, conical, verrucose to felted, white (#fffff), sometimes yellowish (#ffffd8), removable volval remnants on the pileus; margin striate (up to 0.2 R), non-appendiculate; trama white (#ffffff), unchanging. White lamellae (#fffff), truncated lamellulae. Stipe 7–16 cm long, 0.5–2.0 cm wide at the apex., white (#fffff), covered with white (#fffff) fibrils; white (#fffff) context, unchanging; ovoid, fusiform to subglobose basal bulb (1–4-cm diam.); verrucose to conical, white (#fffff) to yellowish (#ffffd8) volval remnants on the stipe base, sometimes arranged in incomplete rings. Subapical to submedian annulus. Spore print not observed. Odor indistinct.

Basidia (40–60 × 12–15 μm), clavate, four-spored. Basidiospores [30/1/1] (9–)9.5–10.8(–12.5) × (7–)7.3–8.5(–9.0) μm, Q = (1.2–)1.24–1.45(–1.47), Qm = 1.32 ± 0.06, broadly ellipsoid to ellipsoid, inamyloid. Volval remnants on the pileus are composed of vertically arranged elements: scattered filamentous hyphae; very abundant inflated cells. The structure of volval remnants on the stipe base is similar to that of volval remnants on the pileus, but with irregularly arranged elements. Clamps are present in all parts of basidioma.

**Distribution:** This species is known to be found in Asia (Imai, 1933; Imai, 1938; Kumar et al., 1990; Yang, 2005; Geml et al., 2006; Geml et al., 2008; Yang, 2015), Europe (Neville and Poumarat, 2004), North America (Geml et al., 2006; Geml et al., 2008), and northeastern and northwestern areas of China (Cui et al., 2018).

**Habitat and distribution:** It is present individually or is scattered in the broad-leaved forests of *B. platyphylla* Suk. Basidioma occurs in summer and autumn.

**Specimens examined:** CHINA. Hebei Province, Chicheng County, Dahaituo Mountains National Nature Reserve, 40.563828 N, 115.798698 E, elev. 1640 m, 22 August 2018, coll. C.L. Hou, J.Q. Li and G.Q. Cheng (L491/BJTC L491).

**Commentary:** *A. muscaria* was described from Europe and represents the type species of *Amanita* (Gilbert, 1940; Gilbert, 1941; Jenkins and Petersen, 1976; Galli, 2001; Neville and Poumarat, 2004; Yang, 2005; Yang, 2015; Cui et al., 2018). Yang and Oberwinkler (1999) conducted a detailed study on *A. muscaria* basidiomal development and anatomy. The morphological description of our specimen is consistent with that given by Cui et al. (2018). In our multi-locus analysis (**Figure 2**), *A. muscaria* was found to be closely related to *A. persicina* (D.T. Jenkins) Tulloss & Gem. These results are consistent with those of Cui et al. (2018). Based on these characters, our specimen BJTC L491 was described as *A. muscaria*. Detailed descriptions of *A. muscaria* can be found in Cui et al. (2018).

#### 3.2.6 *Amanita oberwinkleriana* Zhu L. Yang & Yoshim. Doi, Bull. Natn. Sci. Mus., Tokyo, Ser. B 25 (3): 120 (1999)

**Figure 7:**
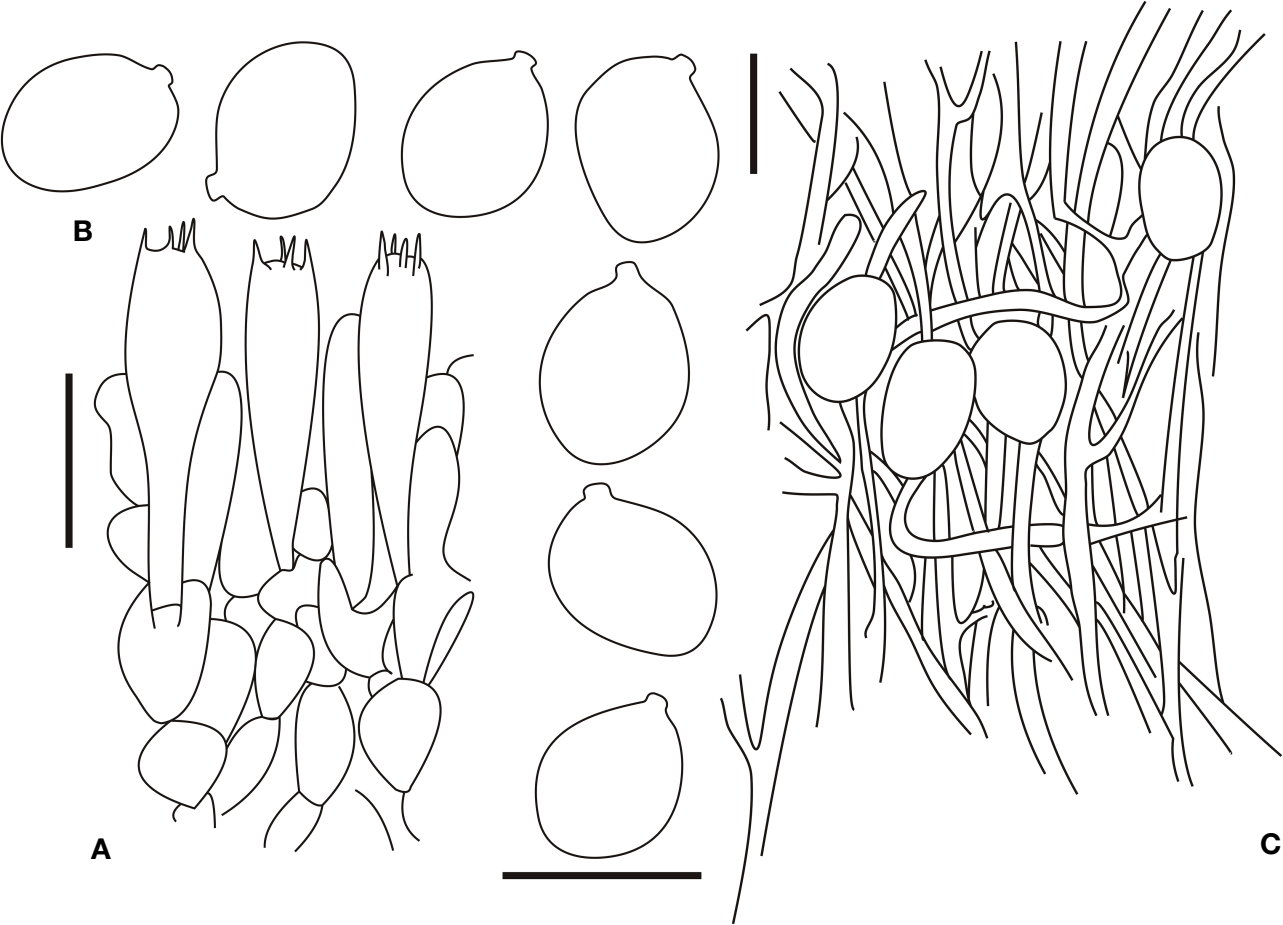
Microscopic characters of *Amanita oberwinkleriana* (BJTC C320). **(A)**. Hymenium and subhymenium; **(B)**. Basidiospores; **(C)**. Longitudinal section of outer part of volval remnants on stipe base. Bars: **(A, B)** = 10 μm, **(C)** = 40 μm.

**Figures 6G–I**, **7**

Basidiomata small- to medium-sized. Pileus 3–8 cm in diam., applanate, no slightly depressed at the center, surface gray-white (#e6e6e6) to white (#ffffff), large, gray-white (#e6e6e6) volval remnants on the pileus (ca. 2–4 (–6)-mm diam.), densely arranged over the disk; margin slightly striate (ca. 0.05–0.15 R), non-appendiculate. Free, white (#ffffff), unchanging, somewhat crowded lamellae; attenuate, plentiful lamellulae. Stipe 8–15 cm long, 0.5–2.0 cm wide at the apex, subcylindric or slightly tapering upward, with the apex slightly expanded, gray-white (#e6e6e6) to white (#ffffff), with minute concolorous squamules; grayish (#e6e6e6), unchanging context; subglobose basal bulb (1.0–2.0-cm diam.), dirty white (#f2f2f2), with upper part covered with verrucose, dirty grayish (#e6e6e6) to dirty white (#f2f2f2) volval remnants arranged in irregular concentric rings. Annulus absent. Spore print not observed. Odor indistinct.

Basidia (28–45 × 8–13 μm), slenderly clavate, four-spored, with clamps, hyaline to pale yellow (#ffffd8); 5–6-μm-long sterigmata, clamped basal septa; basidiospores [80/4/3] (6–)7.0–10(–10.5) × (5–)5.5–6.8(7.5) μm, Q = (1.25–)1.35–1.58(–1.62), Qm = 1.41 ± 0.11, mostly ellipsoid, occasionally broadly ellipsoid, thin-walled, hyaline, pale yellow, smooth, with a small to medium-large apiculus, amyloid. Volval remnants on the stipe base are similar to those on the pileus, but with more abundant filamentous hyphae and fairly abundant vascular hyphae. Clamps are present in all parts of basidioma.

**Distribution:** This species is known to be present in central, eastern, southern, and southwestern China (Yang and Li, 2001; Yang, 2005; Yang, 2015; Cui et al., 2018); India (Bhatt et al., 2003); Japan (Yang and Doi, 1999); and Republic of Korea (Kim et al., 2013a).

**Habitat and distribution:** It is present individually or is scattered in the coniferous forests and mixed coniferous and broad-leaved forests of *Pinus tabuliformis* Carr., *Q. mongolica* Fisch. ex Ledeb., and *P. davidiana* Dode. Basidioma occurs in summer and autumn.

**Specimens examined:** CHINA. Beijing, Hebei Province, Xinglong country, Babaziling village, 40.310326 N, 117.585117 E, alt. 878 m, 22 Aug., 2020, coll. G.Q. Cheng., C.L. Hou.and Y.T. Zhang (C320/BJTC C320); CHINA. Tianjin, Jizhou district, JiulongShan Mountains National Forest Park, 40.152701 N, 117.508008 E, alt. 300 m, 18 Aug., 2019, coll. H. Zhou, X.Y. Shen and Y.T. Zhou (ZH328/BJTC Z328); CHINA. Beijing, Pinggu district, Dongxinzhuang village, 40.560714 N, 116.779865 E, alt. 286 m, 27 Aug., 2020, coll. G.Q. Cheng., C.L. Hou and Y.T. Zhang (C659/BJTC C659); CHINA. Tianjin, Jizhou district, JiulongShan Mountains National Forest Park, 40.151969 N, 117.509612 E, alt. 226 m, 18 Aug., 2019, coll. H. Zhou, X.Y. Shen and Y.T. Zhou (ZH318/BJTC Z318); CHINA. Beijing, Huairou district, Baiquanshan, 40.496637 N, 116.649129 E, alt. 268 m, 17 Aug., 2020, coll. H. Zhou, X.Y. Shen and Y.T. Zhou (ZH862/BJTC Z862); CHINA. Beijing, Yanqing district, Songshan Mountains National Nature Reserve, 40.27 N, 115.57 E, elev. unknown, 11 July 2011, coll. unknown (HMAS 263406); CHINA. Beijing, Mentougou district, Donglingshan Mountains, 40.22 N, 116.50 E, elev. unknown, 29 July., 2013, coll. W.L. Lu (HMAS 253800); CHINA. Beijing, Mentougou district, Donglingshan Mountains, 40.22 N, 116.50 E, elev. unknown, 29 July., 2013, coll. W.L. Lu (HMAS 253801); CHINA. Beijing, Mentougou district, Donglingshan Mountains, 40.22 N, 116.50 E, elev. unknown, 29 July., 2013, coll. W.L. Lu (HMAS 253802); CHINA. Beijing, Mentougou district, Yunmengshan Mountains, 40.22 N, 116.50 E, elev. unknown, 29 July., 2013, coll. T.Z Wei (HMAS 253796).

**Commentary:** Yang and Doi (1999) described *A. oberwinkleriana* from Japan, and it was subsequently reported from China, India, and Republic of Korea (Yang and Li, 2001; Bhatt et al., 2003; Yang, 2005; Kim et al., 2013a; Yang, 2015; Cui et al., 2018). The morphological description of our specimen is consistent with that provided by Yang and Doi (1999). In our multi-locus phylogenetic analysis, 10 specimens clustered together with *A. oberwinkleriana* (HKAS 77330), forming a completely supported clade (BPP = 1.00, MLB = 96%). Based on these characters, our specimens were described as *A. oberwinkleriana*. Of note, the samples from the herbarium specimens (HMAS) exhibited poor sequence quality because of a long time. The multi-locus and nrLSU phylogenetic trees revealed that the branches of these herbarium specimens were longer (**Figures 2**, **S1**).

#### 3.2.7 *Amanita ovalispora* Boedijn, Sydowia 5(3-6): 320 (1951)

**Figure 8:**
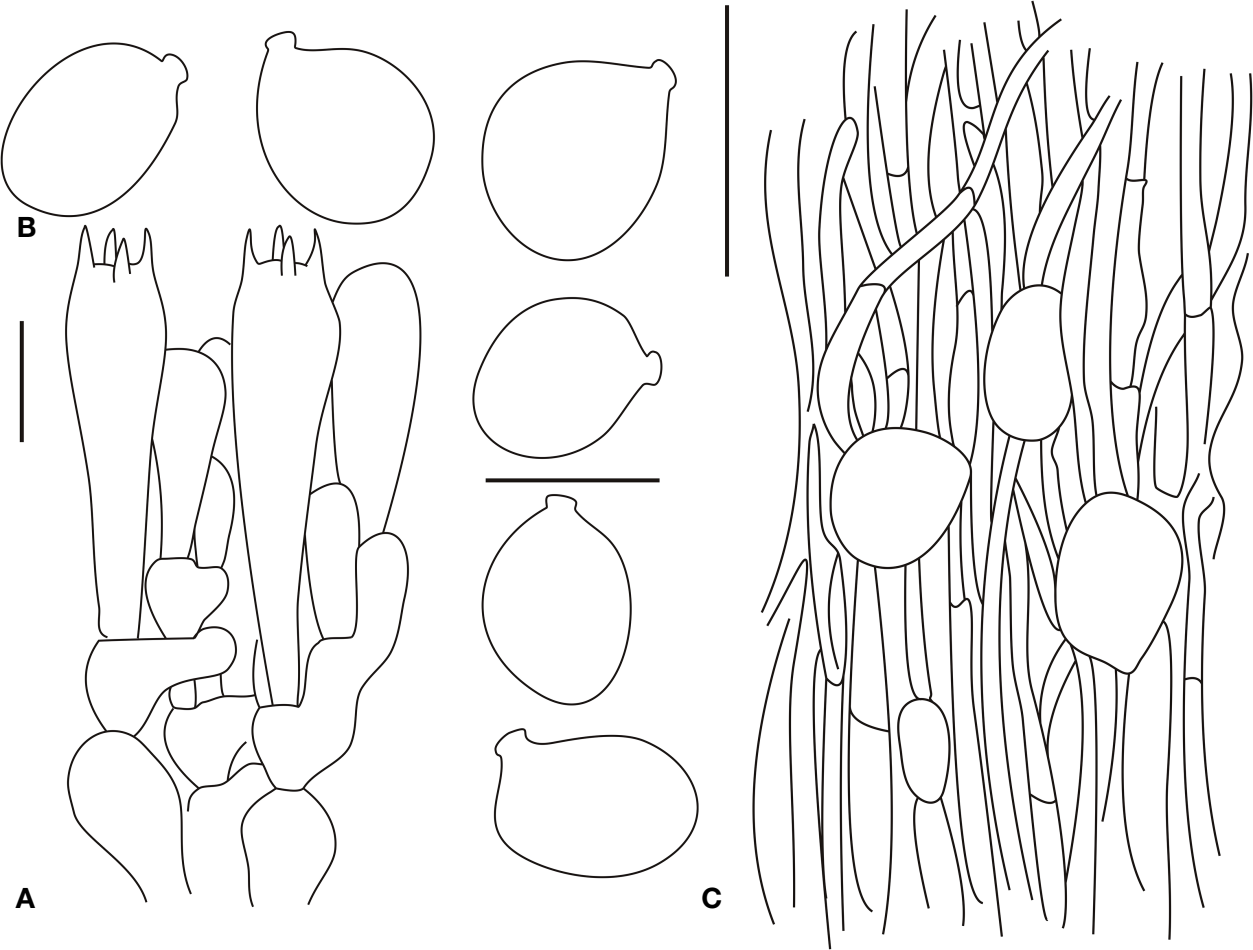
Microscopic characters of *Amanita ovalispora*. (BJTC Z311). **(A)**. Hymenium and subhymenium; **(B)**. Basidiospores; **(C)**. Longitudinal section of outer part of volval remnants on stipe base. Bars: **(A, B)** = 10 μm, **(C)** = 40 μm.

**Figures 6J, K**, **8**

Basidiomata small- to medium-sized. Pileus 3–6 cm in diam., plano-convex to applanate, distinctly umbonate at the center, gray-brown (#75615a) to gray (#808080), absence of volval remnants on the pileus, margin striate (0.2–0.5 R), non-appendiculate; trama white (#ffffff), unchanging. Free, crowded, white (#ffffff) lamellae; white (#ffffff) lamellar edge; truncated, plentiful lamellulae. Stipe 8–16 cm long, 0.5–1.5 cm wide at the apex, subcylindric or slightly tapering upward, with the apex slightly expanded, white (#ffffff) to dirty white (#f2f2f2), glabrous or covered with minute, concolorous fibrils; white (#ffffff), hollow in the center context; absence of basal bulb; volva saccate, membranous, both white (#ffffff) surfaces. Annulus absent. Spore print not observed. Odor indistinct.

Basidia (30–55 × 8–14 μm), clavate, four-spored, basal septa lacking clamps, hyaline; 4–6-μm-long sterigmata; basidiospores [60/2/2] (8–)9.5–11.8(–12.5) × (7–)7.3–9.1(–10.2) μm, Q = (1.15–)1.23–1.49(–1.52), Qm = 1.34 ± 0.10, globose to subglobose, thin-walled, hyaline, smooth, with a small apiculus, inamyloid. Volval remnants on the stipe are composed of longitudinally arranged elements: very abundant filamentous hyphae. Outer surface of the volval remnants on the stipe base is similar to the structure of the outer part, but with more abundant filamentous hyphae; gelatinized inner surface with structure similar to that of the inner part. Clamps are absent in all parts of basidioma.

**Distribution:** This species is known to be present in southern and southwestern China (Yang, 1997; Yang, 2005; Yang, 2015; Cui et al., 2018), and Indonesia (Boedijn, 1951).

**Habitat and distribution**: It is present individually or scattered in the coniferous forests and mixed coniferous and broad-leaved forests of *Pinus tabuliformis* Carr. and *Juglans mandshurica* Maxim., with basidioma occurring in summer and autumn.

**Specimens examined:** CHINA. Tianjin, Jizhou district, JiulongShan National Forest Park, 40.147837 N, 117.510365 E, alt. 174 m, 18 Aug., 2019, coll. H. Zhou, X.Y. Shen and Y.T. Zhang (ZH311/BJTC Z311); CHINA. Beijing, Changping district, Tibiyinshan, 40.317323 N, 116.321293 E, alt. 353 m, 14 Aug., 2019, coll. H H. Zhou, X.Y. Shen and Y.T. Zhang (ZH057/BJTC Z057).

**Commentary:** Boedijn (1951) was first described *A. ovalispora* from Indonesia. Subsequently, Yang (1997) examined the holotype, and described their basidiospores. According to Cui et al. (2018), no sequence of *A. ovalispora* is available from its type locality to delimit this species accurately. The morphological description of our specimens is consistent with that provided by Boedijn (1951). In our multi-locus phylogenetic analysis, two specimens clustered together with *A. oberwinklerana* (HKAS 79625 and HKAS 101406), forming a completely supported clade (BPP = 1.00, MLB = 96%) (**Figure 2**). The nrLSU phylogenetic analysis exhibited topologies similar to those of the multi-locus phylogenetic tree (**Figure S1**). According to these characters, our specimens were described as *A. ovalispora*.

#### 3.2.8 *Amanita subglobosa* Zhu L. Yang, Bibl. Mycol. 170: 18 (1997)

Basidiomata small- to medium-sized. Pileus 4–8 cm in diam., brownish (#a5682a) to dark brown (#68421a); pyramidal to verrucose, white (#ffffff) to yellowish (#ffff9a), removable volval remnants on the pileus; margin striate (0.1–0.4 R), non-appendiculate; trama white (#ffffff), unchanging. White (#ffffff) to cream (#fffeea) lamellae, truncated lamellulae. Stipe 5–15 cm long, 0.5–2 cm wide, white (#ffffff) to dirty white (#f2f2f2); white (#ffffff), unchanging context; subglobose basal bulb (1.5–3.5-cm diam.), with its upper part covered with conical to pulverulent, yellowish (#ffff9a) to brownish (#a5682a) volval remnants, often forming a collar between the limit of the stipe and basal bulb. Subapical to submedian, white (#ffffff), persistent annulus. Spore print not observed. Odor indistinct.

Basidia (35–55 × 10–15 μm), clavate, four-spored. Basidiospores [60/2/2] (7.0–)8.3–11.0 (–13.0) × (6.0–)6.5–9.5(–11.5) μm, Q = (1.05–) 1.13–1.40(–1.60), Qm = 1.29 ± 0.08, broadly ellipsoid to ellipsoid, inamyloid. Volval remnants on the pileus are composed of vertically arranged elements: fairly abundant filamentous hyphae; abundant inflated cells. Clamps are present in all parts of basidioma.

**Distribution:** This species is known to be present in central, northeastern, and southwestern China (Yang, 1997; Yang, 2005; Yang, 2015; Cui et al., 2018); India (Semwal et al., 2007); Republic of Korea (Kim et al., 2013b); and Thailand (Sanmee et al., 2008).

**Habitat and distribution:** It is present solitary or is scattered in the pine, broad-leaved, or mixed forests of *Fagaceae* and *Pinaceae* trees. Basidioma occurs in summer and autumn (Cui et al., 2018).

**Specimens examined:** CHINA. Beijing, Mentougou district, Baihuashan Mountains, 39.52 N, 115.36 E, elev. unknown, 22 Aug., 1964, coll. Y.C. Zong and Q.T. Tao (HMAS 34658); CHINA. Beijing, Mentougou district, Tanzhe Temple, 39.54 N, 116.01 E, elev. unknown, 22 Aug., 1965, coll. Q.M. Ma (HMAS 253798).

**Commentary:** Yang (1997) first described *A. subglobosa* from China. Later, it was reported from India, Republic of Korea, and Thailand (Semwal et al., 2007; Sanmee et al., 2008; Kim et al., 2013b). The morphological description of our specimen is consistent with that provided by Yang (1997). Our multi-locus phylogenetic analysis revealed that a specimen HMAS 253798 clustered together with *A. subglobosa* (HKAS 67914), forming a completely supported clade (BPP = 1.00, MLB = 100%) (**Figure 2**). The nrLSU phylogenetic analysis revealed topologies similar to those of the multi-locus phylogenetic tree (**Figure S1**). Based on these characters, we described our specimen as *A. subglobosa*. In addition, DNA sequences from another herbarium specimens (HMAS 34658) could not be generated. However, we also examined the morphology of these specimens, and the results proved that these specimens were *A. subglobosa*.

#### 3.2.9 *Amanita subjunquillea* S. Imai, Bot Mag (Tokyo) 47: 424 (1933)

**Figure 9:**
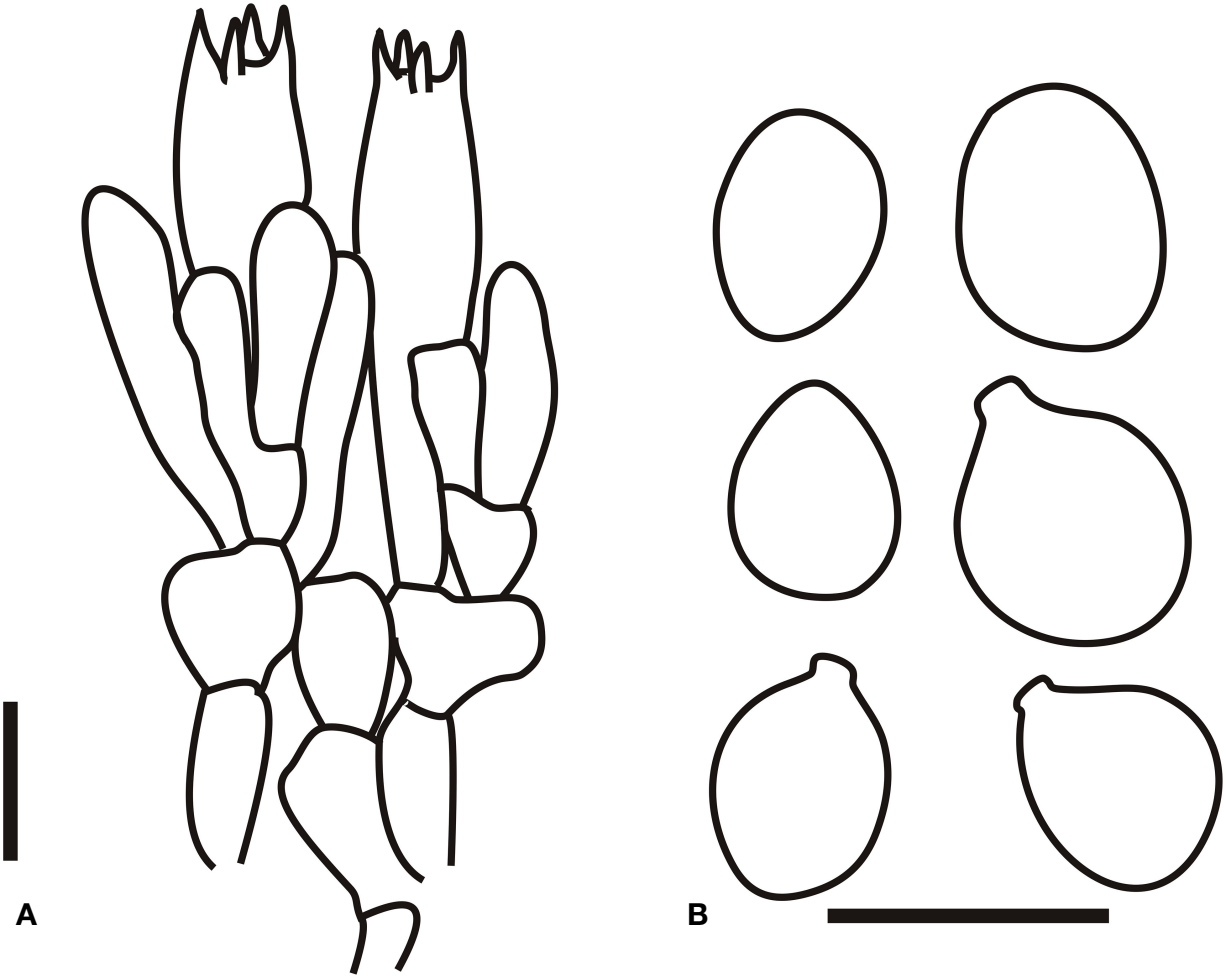
Microscopic characters of *Amanita subjunquillea* (BJTC 217). **(A)**. Hymenium and subhymenium; **(B)**. Basidiospores. Bars: **(A, B)** = 10 μm.

**Figures 6L, M**, **9**

**Synonym:** *Amanita subjunquillea* var. *alba* Zhu L. Yang, Bibl. Mycol. 170: 174 (1997)

Basidiomata small- to medium-sized. Pileus 3–6 cm in diam., globose when young, hemispherical when expanding, later plano-convex to applanate, surface pale yellow (#ffff00) to light yellow (#ffffa1), becoming paler toward the margin; absence of volval remnants on the pileus. Free, white (#ffffff) to cream (#fffdd0), plentiful lamellae. Stipe 4–10 cm long, 0.8–2 cm wide at the apex, subcylindric or slightly tapering upward, with the apex slightly expanded, surface pale yellow (#ffff00) to light yellow (#ffffa1), with silk luster, upper part often covered with pale yellow (#ffff00) floccose to farinose squamules, lower part often covered with white (#ffffff) to pale yellow (#ffff00) floccose squamules; white (#ffffff) to pale yellow (#ffff00) context; marginate, dirty white (#fcfcfc) to grayish (#cccccc) basal bulb (1–2-cm diam.), with upper edge shortly limbate, dirty white (#fcfcfc) to grayish (#cccccc) volval remnants. Annulus absent. Spore print not observed. Odor indistinct.

Basidia (25–55 × 6.5–12 μm), slenderly clavate, four-spored, with clamps, hyaline; 3–5-μm-long sterigmata; basidiospores [60/4/4] (6–)6.5–8.4(–9) × (4–)4.1–6.6(–7.5) μm, Q = (1.02)1.18–1.36(1.51), Qm = 1.34 ± 0.20, mostly ellipsoid, occasionally spherical, thin-walled, hyaline, smooth, with a small apiculus, weakly amyloid. Volval remnants on the pileus are composed of irregularly to subvertically arranged elements: fairly abundant, branching, anastomosing filamentous hyphae; very abundant, globose, subglobose to ellipsoid inflated cells. Clamps are present in all parts of basidioma.

**Distribution:** This species is known to be from central, northern, northeastern, northwestern, and southwestern China (Yang, 1997; Yang, 2005; Yang, 2015; Cai et al., 2016; Cui et al., 2018); India (Bhatt et al., 2003; Bhatt et al., 2007); Japan (Imai, 1933; Imai, 1938; Yang and Doi, 1999; Imazeki et al., 2011); and Republic of Korea (Kim et al., 2013a; Cho et al., 2015).

**Habitat and distribution:** It is present individually or is scattered in coniferous forests and mixed coniferous and broad-leaved forests of *Pinus tabuliformis* Carr., *J. mandshurica* Maxim, and *P. davidiana* Dode., with basidioma occurring in summer and autumn.

**Specimens examined:** CHINA. Beijing, Yanqing district, Songshan Mountains National Nature Reserve, 40.596637 N, 115.849129 E, alt. 648 m, 17 Sep., 2017, coll. C.L. Hou, H. Zhou and J.Q. Li (217/BJTC 217); CHINA. Beijing, Yanqing district, Songshan Mountains National Nature Reserve, 40.316637 N, 115.459129 E, alt. 1102 m, 17 Sep., 2017, coll. C.L. Hou, H. Zhou and J.Q. Li (085/BJTC 085); CHINA. Beijing, Yanqing district, Songshan Mountains National Nature Reserve, 40.517899 N, 115.820799 E, alt. 906 m, 20 Aug., 2018, coll. C.L. Hou, H. Zhou and J.Q. Li (704/BJTC 704); CHINA. Beijing, Yanqing district, Songshan Mountains National Nature Reserve, 40.30024 N, 115.499129 E, alt. 860 m, 17 Sep., 2017, coll. C.L. Hou, H. Zhou and J.Q. Li (033/BJTC 033); CHINA. Beijing, Mentougou district, Donglingshan Mountains, 40.22 N, 116.50 E, elev. unknown, coll. unknown (HMAS 253775); CHINA. Beijing, Yanqing district, Songshan Mountains National Nature Reserve, 40.31 N, 115.45 E, alt. 1216 m, 16 Sep., 2017, coll. C.L. Hou, H. Zhou and J.Q. Li (112/BJTC 112); CHINA. Beijing, Hebei Province, Xinglong country, Changgou, 40.205334 N, 117.625724 E, alt. 878 m, 18 Aug., 2019, coll. G.Q. Cheng., C.L. Hou.and H. Zhou (ZH276/BJTC Z276); CHINA. Beijing, Huairou district, Sunzhazi Village 40.943482 N, 116.507391 E, alt. 789 m, 25 Aug., 2020, coll. C.L. Hou, G.Q. Cheng and Y.T. Zhang (C558/BJTC C558); CHINA. Beijing, Shunyi district, Mulin Town, 40.235317 N, 116.819856 E, alt. 45 m, 16 Aug., 2019, coll. G.Q. Cheng., C.L. Hou.and H. Zhou (ZH172/BJTC Z172).

**Commentary:** Imai (1933) first described *A. subjunquillea* from Japan. Later, it was reported from China, India, and Republic of Korea (Yang, 1997; Bhatt et al., 2003; Yang, 2005; Bhatt et al., 2007; Kim et al., 2013a; Cho et al., 2015; Yang, 2015; Cai et al., 2016; Cui et al., 2018). This species is deadly poisonous (Imazeki et al., 1988; Kawase et al., 1992; Chen et al., 2016; Tang et al., 2016). The morphological description of our specimen is consistent with that provided by Imai (1933). In our multi-locus phylogenetic analysis, nine specimens clustered together with *A. oberwinklerana* (HKAS 75771, HKAS 75770, and HKAS 75773), forming a completely supported clade (BPP = 1.00, MLB = 99%) (**Figure 2**). The nrLSU phylogenetic analysis showed topologies similar to those of the multi-locus phylogenetic tree (**Figure S1**). Based on these characters, our specimens were described as *A. subjunquillea*.

#### 3.2.10 *Amanita vaginata* var. *vaginata* (Bull.) Lam., Encycl. Méth. Bot. (Paris) 1(1): 109 (1783)

**Figures 6N, O**

**Basionym:** *Agaricus vaginatus* Bull., Herb. Fr. (Paris) 3: tab. 98 (1783) [1782-83].

Basidiomata small- to medium-sized. Pileus 3–7 cm in diam., somewhat umbonate, grayish (#a6a6a6) to gray (#b3b3b3); volval remnants on the pileus absent or retained as white (#ffffff) patches; margin striate (0.1–0.3 R), non-appendiculate. White (#ffffff) lamellae; truncated lamellulae. Stipe 5–10 cm long, 0.5–2.0 cm wide at the apex., white (#ffffff) to dirty white (#fcfcfc), glabrous or covered with fibrous, grayish (#a6a6a6) to gray (#b3b3b3) fibrils; absence of basal bulb; volval remnants on the stipe base saccate, outer surface white (#ffffff) to dirty white (#fcfcfc), inner surface white (#ffffff). Annulus absent. Spore print not observed. Odor indistinct.

Basidia (48–60 × 13–18 μm), clavate, four-spored. Basidiospores [60/3/3] (9.2–) 9.5–11.2 (–13.0) × (8.0–) 9.0–10.9 (–13.0) μm, Q = (1.0–)1.02–1.08 (–1.10), Qm = 1.01 ± 0.03, globose to subglobose, inamyloid. Clamps are absent in all parts of basidioma.

**Distribution:** This species is known to be from Asia (Imai, 1933; Imai, 1938; Tulloss et al., 2001; Yang, 2005; Imazeki et al., 2011; Kim et al., 2013b; Cui et al., 2018), North America (Coker, 1917; Thiers, 1982; Jenkins, 1986; Tulloss et al., 1995), and Europe (Horak, 1968; Consiglio, 2000).

**Habitat and distribution:** It is present individually or is scattered in broad-leaved forests of *Fagaceae*. Basidioma occurs in summer and autumn.

**Specimens examined:** CHINA. Beijing, Yanqing district, Songshan Mountains National Nature Reserve, 40.512712 N, 115.817818 E, elev. 555 m, 26 August 2018, coll. C.L. Hou, J.Q. Li and H. Zhou (682/BJTC 682); CHINA. Beijing, Yanqing district, Songshan Mountains National Nature Reserve, 40.512154 N, 115.817489 E, elev. 901 m, 26 August 2018, coll. C.L. Hou, J.Q. Li and H. Zhou (677/BJTC 677); CHINA. Beijing, Huairou district, Shangdian, 40.927121 N, 116.697837 E, elev. 681 m, 19 August 2019, coll. H. Zhou, X.Y. Shen and R.T. Zhang (ZH521/BJTC Z521); CHINA. Beijing, Mentougou district, Donglingshan Mountains, 39.55 N, 116.24 E, elev. unknown, coll. unknown (HMAS 253281); CHINA. Beijing, Miyun district, 40.22 N, 116.5 E, elev. unknown, August 1998, coll. X.L. Mao (HMAS 78411); CHINA. Beijing, Mentougou district, Donglingshan Mountains, 39.55 N, 116.24 E, elev. unknown, 19 August 1998, coll. H.A. Wen and S.X. Sun (HMAS 75237).

**Commentary:** *A. vaginata* var. *vaginata* is characterized by a gray pileus with striations at the margin, a white stipe lacking an annulus, and globose to subglobose basidiospores (9.0–13.0 (–14.0) μm) (Lange, 1935; Huijsman, 1959; Bas, 1967; Horak, 1968; Jenkins, 1986; Breitenbach and Kränzlin, 1995; Tulloss et al., 1995). In our multi-locus phylogenetic analysis, four specimens clustered together with *A. vaginata* var. *vaginata* (LAF 024482, type), forming a completely supported clade (BPP = 1.00, MLB = 99%) (**Figure 2**). The nrLSU phylogenetic analysis displayed topologies similar to those of the multi-locus phylogenetic tree (**Figure S1**). Based on these characters and results of phylogenetic analyses, our specimens were described as *A. vaginata* var. *vaginata*. Detailed descriptions, line drawings, and images of *A. vaginata* var. *vaginata* are available in Yang (2015). Unfortunately, DNA sequences could not be generated from two herbarium specimens (HMAS 78411 and HMAS 75273). However, on examining the morphology of these specimens, we confirmed that these specimens were *A. vaginata* var. *vaginata.*

#### 3.2.11 *Amanita virosa* Bertillon, Dict. Encycl. Sci. Médic.: 497 (1866)

**Replaced synonym:** *Agaricus virosus* Fr., Epicr. syst. mycol. (Upsaliae): 3 (1838) [1836-1838]; *Agaricus virosus* Sowerby, Col. fig. Engl. Fung. Mushr. (London) 3: tab. 407 (1809).

Basidiomata small- to medium-sized. Pileus 5–10 cm in diam., umbonate at the center, white (#ffffff), often cream (#fffdd0) at the center; absence of volval remnants on the pileus; margin non-striate, non-appendiculate; white (#ffffff), unchanging trama. White (#ffffff) lamellae; attenuate lamellulae. Stipe 8–13 cm long, 0.7–2.0 cm wide, white (#ffffff), covered with concolorous squamules; white (#ffffff), unchanging context; Subapical, white (#ffffff), persistent annulus. Spore print not observed. Odor not observed.

Basidia (30–50 × 10–12 μm), clavate, four-spored. Basidiospores [60/2/2] (7.5–)8.0–11.0(–12.0) × (7.0–)8.0–10.0(–11.0) μm, Q = (1.00–)1.05–1.15 (–1.21), Qm = 1.05 ± 0.05, globose to subglobose, amyloid. Volval remnants on the stipe base are composed of longitudinally to irregularly arranged elements: very abundant to nearly dominant filamentous hyphae; scarce to scattered inflated cells. Clamps are absent in all parts of basidioma.

**Distribution:** This species is known to be present in Europe (Konrad and Maublanc, 1924; Chiusa, 2000; Contu, 2000; Neville and Poumarat, 2004) and East Asia (Imai, 1933; Imai, 1938; Zhang et al., 2010; Imazeki et al., 2011; Li et al., 2015; Yang, 2015; Cai et al., 2016; Cui et al., 2018).

**Habitat and distribution:** It is present solitary or is scattered on soil in the subtropical to temperate forests of Fagaceae and Pinaceae. Basidioma occurs in summer and autumn (Cui et al., 2018).

**Specimens examined:** CHINA. Beijing, Miyun district, 40.22 N, 116.50 E, elev. unknown, 4 August 1987, coll. Y.C. Zong (HMAS 40501); CHINA. Beijing, Miyun district, 40.22 N, 116.50 E, elev. unknown, 29 August 1987, coll. Y.C. Zong (HMAS 40530).

**Commentary:** *A. virosa* is widely distributed across Europe and temperate to subtropical Asia (Contu 2000; Chiusa, 2000; Neville and Poumarat, 2004; Zhang et al., 2010; Li et al., 2015; Yang, 2015; Cai et al., 2016; Cui et al., 2018). The morphological description of our specimen is consistent with that provided by Cui et al. (2018). Unfortunately, we could not obtain sequence data from any of the herbarium specimens (HMAS 40501 and HMAS 40503). We also examined the morphology of these specimens, and the results proved that these specimens were *A. virosa.*

#### 3.2.12 *Amanita yanshanensis* H. Zhou & C. L. Hou, sp. nov.

**Figure 10:**
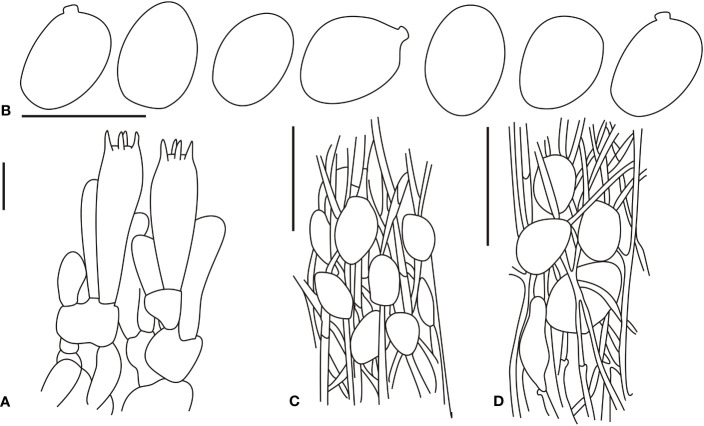
Microscopic characters of *Amanita yanshanensis* (type, BJTC Z049). **(A)**. Hymenium and subhymenium; **(B)**. Basidiospores; **(C)**. Longitudinal section of outer part of volval remnants on stipe base; **(D)**. longitudinal section of inner part of volval remnants on stipe base. Bars: **(A, B)** = 10 μm, **(C, D)** = 40 μm.

**Figures 3E, F**, **10**

**MycoBank: MB 847661**

**Etymology:** The epithet “*yanshanensis*” refers to the locality where the type specimen was collected.

**Type:** CHINA. Beijing, Changping district, Yanshou temple, 40.373152 N, 116.322892 E, alt. 268 m, 14 Aug., 2019, coll. H.Z., X.Y.S.and Y.T.Z. (ZH049/BJTC Z049).

Basidiomata small- to medium-sized. Pileus 4–8 cm diam., sub-hemisphere when young, hemispherical when expanding, later convex, plano-convex to applanate, with an umbo at the center; surface gray-white (#e6e6e6) when young, then gray-white (#e6e6e6) to dark grayish red (#755a5a), large, dark grayish red (#755a5a) to very dark (mostly black) red (#1f1818), or very dark grayish red (#4a3939) volval remnants on the pileus (ca. 2–6 mm diam.), densely arranged over the disk; margin slightly striate (ca. 0.05–0.2 R), non-appendiculate; trama white (#ffffff), unchanging. Free, white (#ffffff), unchanging, somewhat crowded lamellae; attenuate, plentiful lamellulae. Stipe 7–12 cm long, 0.5–1.5 cm wide at the apex, subcylindric or slightly attenuate upward, surface white (#ffffff) to gray-white (#e6e6e6), with silk luster, upper part often covered with gray-white (#e6e6e6) to gray-brown (#755a5a) floccose to farinose squamules, lower part often covered with gray-brown (#755a5a) to dark-brown (#1f1818) verrucose, floccose squamules; white (#ffffff) context; subglobose to clubbed, dirty white (#f2f2f2) to white (#ffffff) basal bulb (1–2 cm diam.); floccose to felted, dark grayish red (#755a5a) to dark (mostly black) red (#1f1818) volval remnants, arranged irregularly or in incomplete belts or rings on the stipe base. Apical, subapical to fugacious, thin annulus, with cream color (#fffeea) and silk luster on the upper surface, a matte cream-colored (#fffeea) lower surface with floccose to farinose warts. Spore print not observed. Odor indistinct.

Bilateral lamellar trama. Mediostratum 20–50 μm wide, composed of abundant ellipsoid to clavate inflated cells (45–60 × 20–40 μm); abundant, 2–6-μm-wide filamentous hyphae; scarce vascular hyphae. Lateral stratum is composed of abundant subfusiform to ellipsoid inflated cells (20–40 × 5–15 μm), diverging at an angle of ca. 30–45° to the mediostratum; abundant, 2–5-μm-wide filamentous hyphae. A 30–55-μm-thick subhymenium, with two to three layers of subglobose to ellipsoid or irregular cells (8–15 × 5–10 μm). Basidia (25–40 × 6–12 μm), slenderly clavate, four-spored, with clamps, hyaline to pale yellow (#ffffd8); 5–6-μm-long sterigmata; basidiospores [120/5/4] 6.0–8.0(–9.0) × 4.0–5.5(–6.0) μm, Q = 1.24–1.45(–1.57), Qm = 1.40 ± 0.12, mostly ellipsoid, occasionally broadly ellipsoid, thin-walled, hyaline, pale yellow, smooth, occasionally with a small to medium-large apiculus, amyloid; sterile lamellar edge, composed of subglobose to ellipsoid inflated cells (20–40 × 10–30 μm), single and terminal or in chains of 2–3, thin-walled, hyaline; abundant, 2–5-μm-wide filamentous hyphae, irregularly arranged or ± running parallel to the lamellar edge. Pileipellis 100–200-μm thick, gelatinized upper layer (25–100-μm thick), composed of subradially to somewhat interwoven, thin-walled, colorless, 2–7-μm-wide filamentous hyphae; lower layer (70–100-μm thick) composed of radially and compactly arranged, pale yellow (#ffffd8), 4–8 (–10)-μm-wide filamentous hyphae; scarce vascular hyphae. Volval remnants on the pileus are composed of inflated cells and filamentous hyphae, more or less arranged in erect chains; abundant to dominant, subglobose, ovoid to ellipsoid, clavate or sphaeropedunculate inflated cells (up to 30 × 25 μm), with hyaline to pale yellow (#ffffd8) vacuolar pigments; septa with clamps; inner part of volval remnants on the pileus often with conspicuous vascular hyphae. Volval remnants on the stipe base are similar to those on the pileus, but with more abundant inflated cells. Stipe trama composed of longitudinally arranged, long clavate terminal cells (50–300 × 15–40 μm); scattered to abundant, 5–10-μm-wide filamentous hyphae; scarce vascular hyphae. Annulus is composed of radially arranged elements: abundant, subglobose, fusiform to ellipsoid, hyaline, thin-walled inflated cells (15–25 × 8–15 μm); abundant, hyaline, thin-walled, 2–5-μm-wide filamentous hyphae. Clamps are present in all parts of basidioma.

**Habitat and distribution:** This species is scattered in the broad-leaved forests of *Castanea mollissima* Blume., with basidioma occurring in summer and autumn.

**Additional specimens examined:** CHINA. Beijing, Changping district, Yanshou Temple, 40.368272 N, 116.321008 E, alt. 234 m, 17 Aug., 2020, coll. H. Zhou, X.Y. Shen and X.B. Huang (ZH820/BJTC Z820); CHINA. Beijing, Changping district, Yanshou temple, 40.368309 N, 116.321011 E, alt. 227 m, 17 Aug., 2020, coll. H. Zhou, X.Y. Shen and X.B. Huang (ZH819/BJTC Z819); CHINA. Beijing, Changping district, Yanshou temple, 40.368654 N, 116.322574 E, alt. 216 m, 17 Aug., 2020, coll. H. Zhou, X.Y. Shen and X.B. Huang (ZH815/BJTC Z815); CHINA. Beijing, Changping district, Yanshou temple, 40.371961 N, 116.321044 E, alt. 245 m, 17 Aug., 2020, coll. H. Zhou, X.Y. Shen and X.B. Huang (ZH824/BJTC Z824); CHINA. Beijing, Changping district, DaYangShan Mountains National Forest Park, 40.308138 N, 116.42437 E, alt. 245 m, 25 July, 2020, coll. H. Zhou, Y.T. Zhang.and X.B. Huang (ZH760/BJTC Z760); CHINA. Beijing, Changping district, DaYangShan Mountains National Forest Park, 40.308003 N, 116.425251 E, alt. 265 m, 14 Aug., 2019, coll. H. Zhou, X.Y. Shen and Y.T. Zhang (ZH083/BJTC Z083); CHINA. Beijing, Pinggu district, Dongxinzhuang village, 40.294008 N, 117.051816 E, alt. 198 m, 19 Aug., 2020, coll. G.Q. Cheng, C.L. Hou.and Y.T. Zhang (C182/BJTC C182); CHINA. Beijing, Changping district, Yanshou temple, 40.373152 N, 116.322892 E, alt. 268 m, 14 Aug., 2019, coll. H. Zhou, X.Y. Shen and X.B. Huang (ZH049/BJTC Z049).

**Commentary:** *A. yanshanensis* is well circumscribed by its gray-white to dark grayish red pileus densely arranged with pyramidal, subverrucose to subconical, floccose volval remnants on the stipe base arranged irregularly or in incomplete belts or rings, and mostly ellipsoid, occasionally broadly ellipsoid basidiospores (6.0–9.0 × 4.0–6.0 μm). Furthermore, it is associated with the trees of *Fagaceae* (*Castanea mollissima*).

This species belongs to section *Validae* and is closely related to *A. spissacea* on the multi-locus phylogenetic tree (**Figure 2**). In the single loci phylogenetic trees of both nrITS and nrLSU, *A. yanshanensis* clustered into an independent clade (**Figures S1**, **S4**). Moreover, basidiomata of *A. yanshanensis* with a gray-brown to gray pileus are also comparable with those of *A. spissacea* and *A. fritillaria* (Sacc.) Sacc. However, *A. fritillaria* has a basal bulb covered with conical, blackish, dark-gray to gray-brown volval remnants and a dirty white to gray annulus (Corner and Bas, 1962; Kumar et al., 1990; Yang, 2005; Yang, 2015; Cui et al., 2018). *A. spissacea* has a stipe covered with grayish to brownish squamules, pulverulent to floccose, a grayish annulus, and wider basidiospores (7.0–9.5× 6.0–7.5 μm) than *A. yanshanensis* (Imai, 1933; Imai, 1938; Hongo, 1959; Imazeki and Hongo, 1987; Imazeki et al., 1988; Imazeki et al., 2011; Cui et al., 2018).

## 4 Discussion

In our study, the molecular phylogenetic analyses further supported the delineation of *Amanita* into two subgenera, namely, subgen. *Amanita* Pers. and *Amanitana* (E.-J. Gilbert) E.-J. Gilbert, as suggested by Cui et al. (2018) (**Figures 2**, **S1**). Based on the macroscopic morphology and the preliminary comparison of the original sequence we obtained, the sections where the specimens were located were initially known. Therefore, the sequence information of subgen. *Lepidella* Beauseigneur and sections *Amarrendiae* (Bougher & T. Lebel) Zhu L. Yang, Y.Y. Cui, Q. Cai & Ling Ping Tang, *Arenariae* Zhu L. Yang, Y.Y. Cui & Q. Cai, *Amidella* (J. E. Gilbert) Konrad & Maubl., and *Strobiliformes* Singer ex Q. Cai, Zhu L. Yang & Y.Y. Cui were not included in the final phylogenetic analyses. Of them, species in sections *Amarrendiae* and *Arenariae* have not been found in China. Cui et al. (2018) mentioned that, for a better understanding of the range of variation in characters, new species should be described based on several specimens, but the technology of molecular systematics used in the present study improved the accuracy of our description of species. As specified in the results, the quality of sequences of these old herbarium specimens (HMAS 283800, HMAS 253802, HMAS 263406, and HMAS 253796) identified as *A. oberwinkleriana* was not good, but its systematic position and combined morphology could still be somewhat helpful in specimen identification. In addition, we could only depend on morphological observations for identifying these specimens as their sequences could not be obtained because of the poor condition of their basidiomata.

In this study, three new species belonged to three sections under the subgenera *Amanita* and *Amanitana*, namely, sections *Amanita* (*A. boreqalis*), *Caesareae* (*A. brunneola*), and *Validae* (*A. yanshanensis*). Section *Amanita* is distinguished by basidioma with agarose; a pileus with persistent volval remnants, a pileal margin striate; truncated lamellae; presence of a basal bulb; and inamyloid basidiospores (Cui et al., 2018). Many species in this section produced neuropsychotoxins (Wieland, 1973; Yang, 2005; Chen et al., 2016). Studies have identified 27 taxa, comprising 23 species, 2 varieties, and 2 forms, in China (Cui et al., 2018; Su et al., 2022). Previous studies have reported 21 species of section *Caesareae* (Cui et al., 2018; Mu et al., 2021). The species belonging to section *Validae* were characterized by a pileal margin non-striae and non-appendiculate; annulus membranous, dominance of filamentous hyphae; volval remnants often as verrucae, warts, flocci, patches, or occasionally as short limb; amyloid basidiospores; and absence of clamps. To date, 18 taxa have been identified from this section (Cui et al., 2018).

Studies have found and recorded 169 *Amanita* species in China, with most of them being concentrated in the southwest, northwest, and south of China, including Yunnan, Guangdong, Heilongjiang, Liaoning, and Hunan provinces (Yang, 1994; Yang, 1997; Yang et al., 2004; Yang, 2005; Deng et al., 2014; Li and Cai, 2014; Ariyawansa et al., 2015; Li et al., 2015; Yang, 2015; Cai et al., 2016; Deng et al., 2016; Liu et al., 2017; Cui et al., 2018; Su et al., 2022). Records of *Amanita* species in Yanshan Mountains, northern China, are few. In this study, 12 *Amanita* species from Yanshan Mountains were recognized. Of them, 10 species or approximately 83% of the species were new or recorded for the first time in this area. Therefore, accelerating the discovery and description of *Amanita* species by using both morphological and molecular approaches in this area is necessary.

Literature review revealed that 14 taxa of *Amanita* were identified from Yanshan Mountains. Three species were identified by Yang (2004): *A. parvipantherina* Zhu L. Yang, M. Weiss & Oberw (HMKS 32350) and *A. griseofolia* Zhu L. Yang (HMKS 22610) collected from Tanzhe Temple in Beijing and *A. subjunquillea* var. *alba* Zhu L. Yang (HMKS 35536) collected from Beishicheng in Beijing. *A. vaginata* var. *vaginata* was collected from Donglingshan Mountains, Beijing (Cui et al., 2018). The morphology of HMKS 75237 was consistent with that of *A. vaginata* var. *vaginata*, but we could not obtain the DNA sequence from the specimen. The specimen HMAS 26491 was collected from Baihuashan Mountains, Beijing, and was originally identified as *A. subglobosa* Zhu L. Yang. However, the results of morphological and phylogenetic analyses conducted in the present study were inconsistent with the original identification. We tentatively named this specimen as *Amanita* sp. because of the poor status of the specimen and await the subsequent collection of additional specimens for further study. In addition, some *Amanita* species have been recorded in the literature even in the absence of any detailed information about the specimen, and so, the accuracy of the information about these species needs to be further verified by collecting specimens and obtaining molecular data. These species with only distribution records available are as follows. *A. caesarea*, *A. inaurata* Gillet, and *A. yuaniana* are distributed in Wulingshan Mountains, Hebei Province (Wang et al., 2005); *A. flavoconia* Alk., *A. phalloides* (Vaill. ex Fr.) Link, and *A. subjunquillea* are distributed in Dahaituo Mountains National Nature Reserve, Hebei Province (Wu et al., 2017); *A. orientigemmata* is distributed in Songshan Mountains National Nature Reserve, Beijing (Wu et al., 2020); *A. verna* Bull ex Lam. is distributed in Badaling Forest Park, Beijing (Zhang et al., 2017); and *A. panterina* (DC. ex Fr.) Schrmm is distributed in Dayangshan National Forest Park, Beijing (Chen et al., 2006). Therefore, specimens of *Amanita* spp. must be more extensively collected from the Yanshan Mountains region to improve the study of their biodiversity.

*Amanita* deserves special attention because of its unique research and popular science education value. However, because some *Amanita* species are similar in morphology and color, distinguishing them in the field is difficult. Moreover, many casualties have been caused through mistakenly consumed poisonous *Amanita* species in many places in China (Li et al., 2020; Li et al., 2021a; Li et al., 2021b). The China Center for Disease Control and Prevention reported eight incidents of mushroom poisoning in Beijing in 2020, with 23 people poisoned. Seven incidents of mushroom poisoning were reported from Hebei Province around Beijing, with 33 people poisoned. In these incidents, the main species causing poisoning were *A. rimosa*, *A. subjunquillea*, and *A. oberwinkleriana* (Li et al., 2021a; Li et al., 2021b). The present study is the initial report on *Amanita’s* biodiversity in the region of Yanshan Mountains, including northern part of Beijing, and Tianjin and Hebei provinces. Given the large area of North China and its diverse forest types, many more *Amanita* species may be discovered in this region in future.

## 5 Conclusions

In this research, 20 *Amanita* specimens deposited in Chinese herbaria and 36 newly collected specimens from North China were studied based on the results of morphological and phylogenetic analyses. In total, 12 phylogenetic species were found. Of them, three species were described as new species, namely *A. borealis* sp. nov., *A. brunneola* sp. nov., and *A. yanshanensis* sp. nov. Furthermore, nine known species were identified, namely, *A. caesareoides*, *A. chiui*, *A. muscaria*, *A. oberwinklerana*, *A. ovalispora*, *A. subglobosa*, *A. subjunquillea*, *A. vaginata* var. *vaginata*, and *A. virosa*. Our results underscore that China has a very high biodiversity of *Amanita* species and that additional studies are required to completely determine the exact number of species. It plays a crucial role in *Amanita* toxin research and ecological conservation. This study investigated the areas where *Amanita* species-related research is lacking. The study attempted to better understand *Amanita* distribution and thus contribute to related research. This study improves the knowledge regarding the species diversity of *Amanita* in Yanshan Mountains and provides new data for the macrofungal systematics, toxin research, and diversity and ecological studies of *Amanita* in subsequent studies.

## Data availability statement

The datasets presented in this study can be found in online repositories (www.treebase.org, study S41229). The NCBI accession number(s) can be found in the article.

## Author contributions

HZ wrote the manuscript, conducted phylogenetic analysis and morphological observations; MG conducted phylogenetic analysis and morphological observations, conducted the experiments; LZ conducted phylogenetic analysis and morphological observations conducted the experiments; HY conducted phylogenetic analysis and morphological observations; XS conducted phylogenetic analysis and morphological observations; YG conducted the experiments; CH conceived and designed the study. All authors contributed to the article and approved the submitted version.
