# Supplementary material for: Diversity and taxonomy of the genus Amanita (Amanitaceae, Agaricales) in the Yanshan Mountains, Northern China
Source: Front Plant Sci. 2023 Sep 14;14:1226794. doi: 10.3389/fpls.2023.1226794 (PMC10539691; doi:10.3389/fpls.2023.1226794)
Supplement: Supplementary file 1 [file DataSheet_1.docx]

Supplementary Material

Diversity and taxonomy of the genus *Amanita* (Amanitaceae, Agaricales) in the Yanshan Mountains, Northern China

Hao Zhou^1,†^, MeiJun Guo^1,†^, Lan Zhuo^1^, HuiFang Yan^1^, XiaoNan Sui^1^, Yue Gao^1^, ChengLin Hou^1,*^

*** Correspondence:** ChengLin Hou chenglin-hou@cnu.edu.cn

# Supplementary Figures

## Supplementary Figures


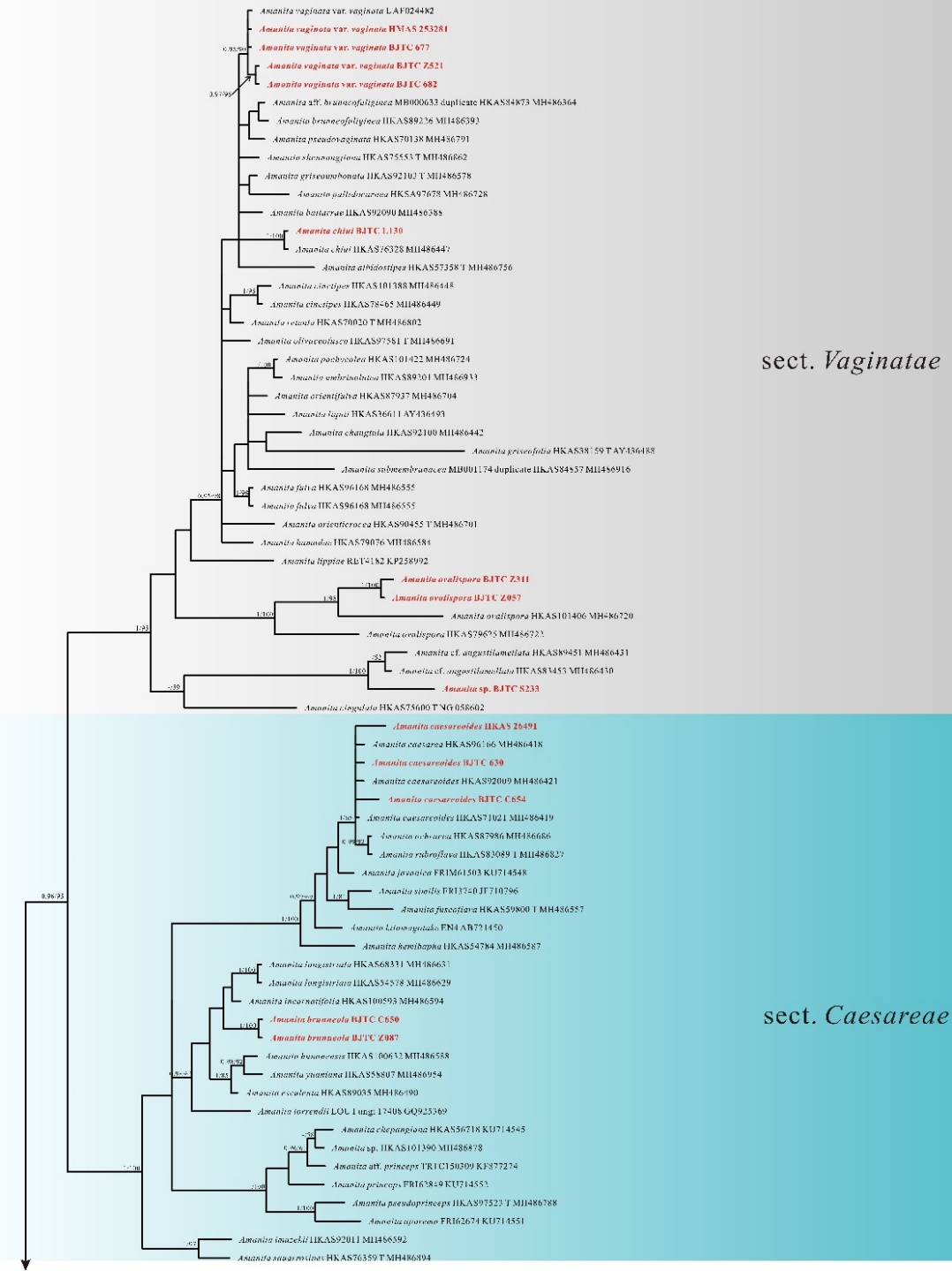


**Supplementary Figure S1. The nrLSU phylogenetic tree obtained from the Bayesian analysis.**

Numbers above branches represent strongly and moderately support (pp≥0.95 and/or MLB≥50%). Numbers above branches are Bayesian posterior probability (pp) values and maximum likelihood bootstrap (MLB). The red font indicates the position of newly obtained sequences. Accession numbers of sequences information used are indicated on the Figure S1. The length of the aligned dataset was 790-bp long.

**
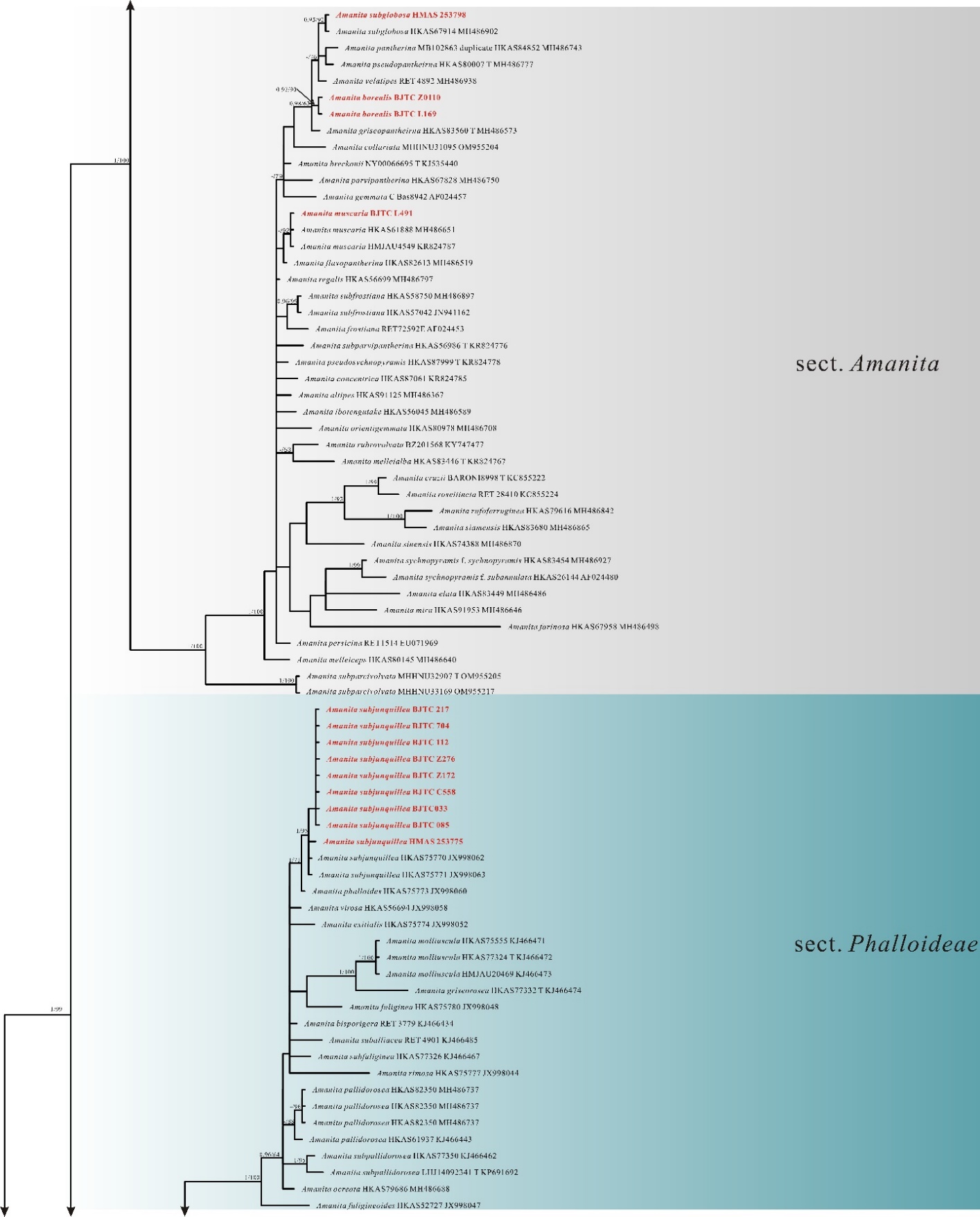
**

**Supplementary Figure S1 Continued**


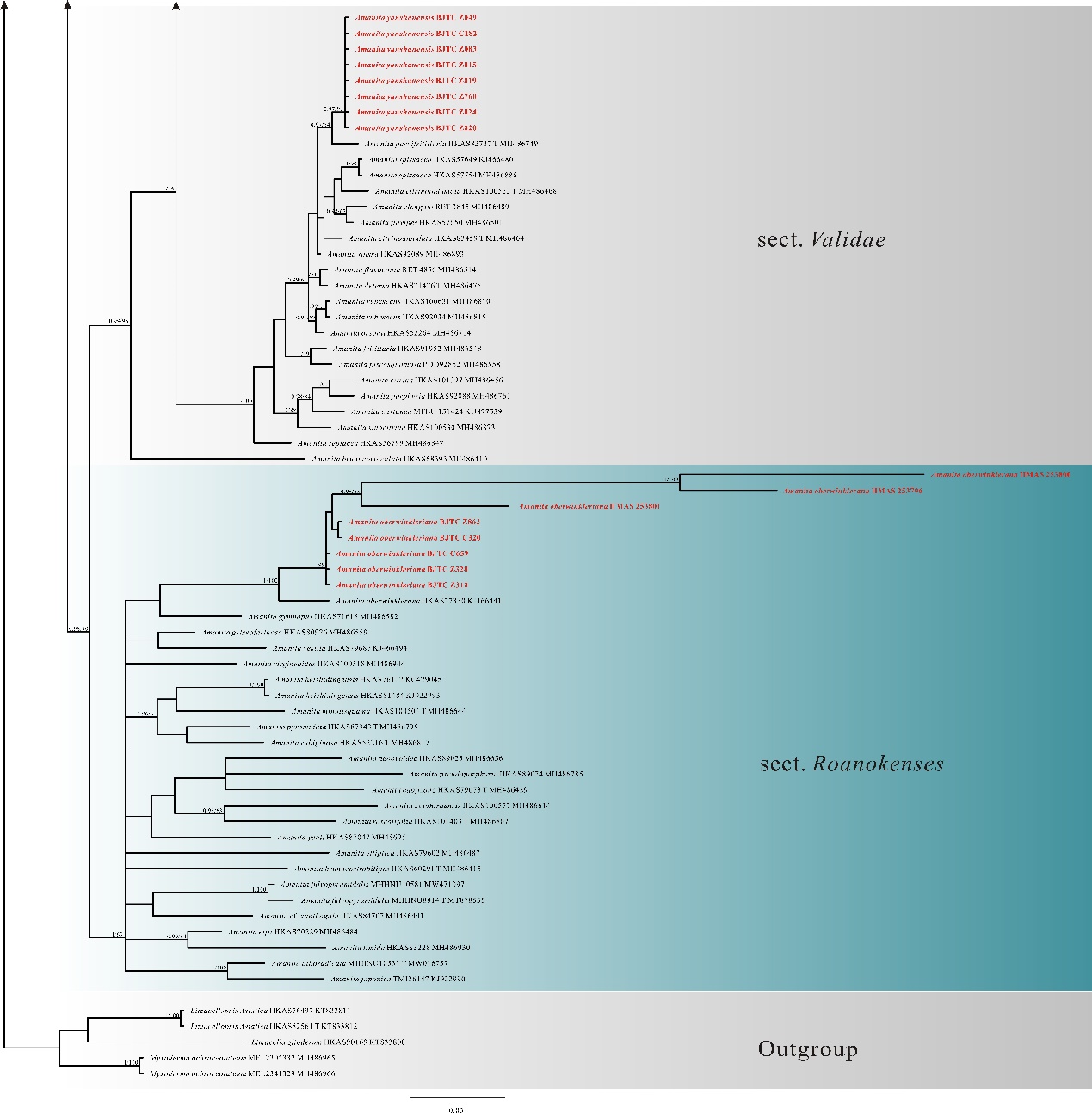


**Supplementary Figure S1 Continued**


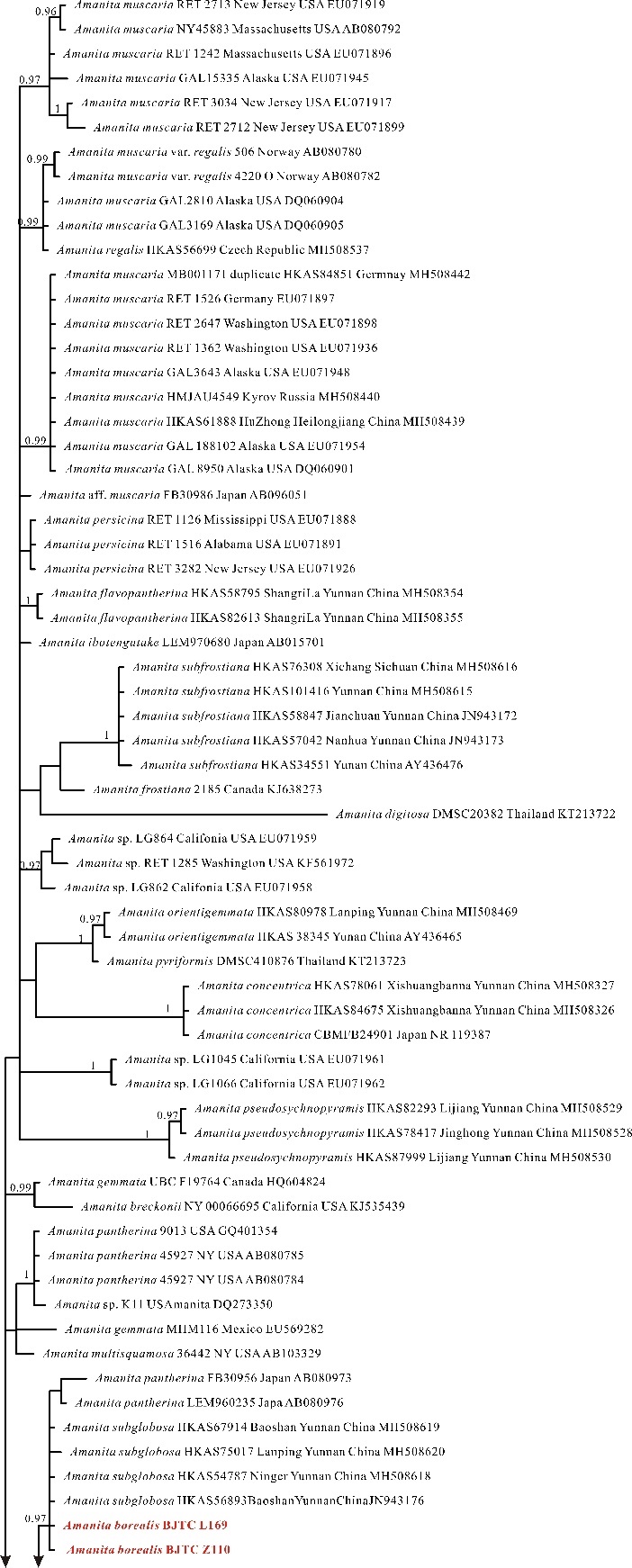


**Supplementary Figure S2 Phylogeny of *Amanita* sect. *Amanita* inferred from the ITS sequences using the Bayesian analysis.**

Bayesian posterior probability (pp) values over 0.95 are shown above or beneath individual branches. New species are highlighted in red. Accession numbers of sequences information used are indicated on the Figure S2. The length of the aligned dataset was 506-bp long.


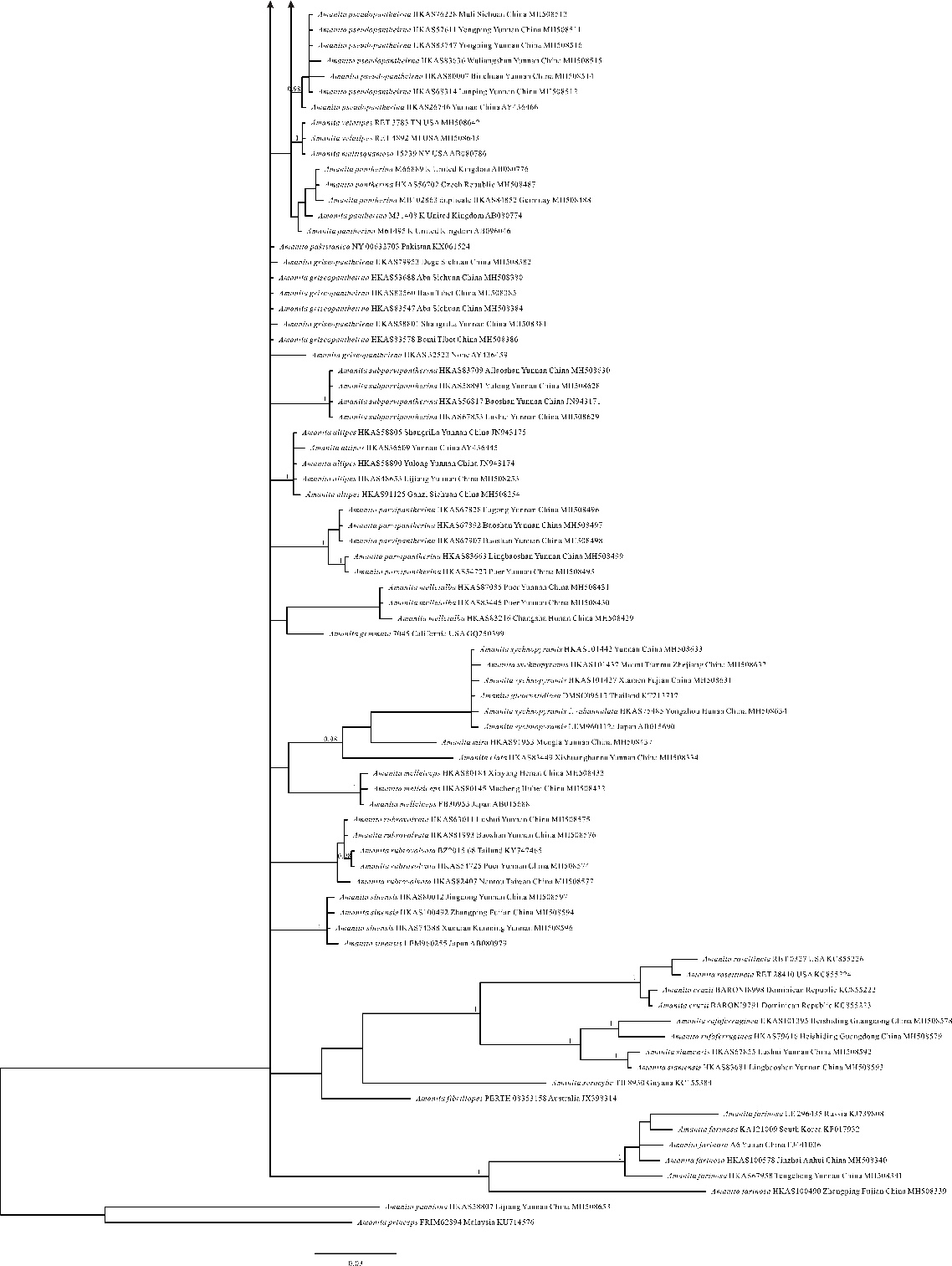


**Supplementary Figure S2 Continued**


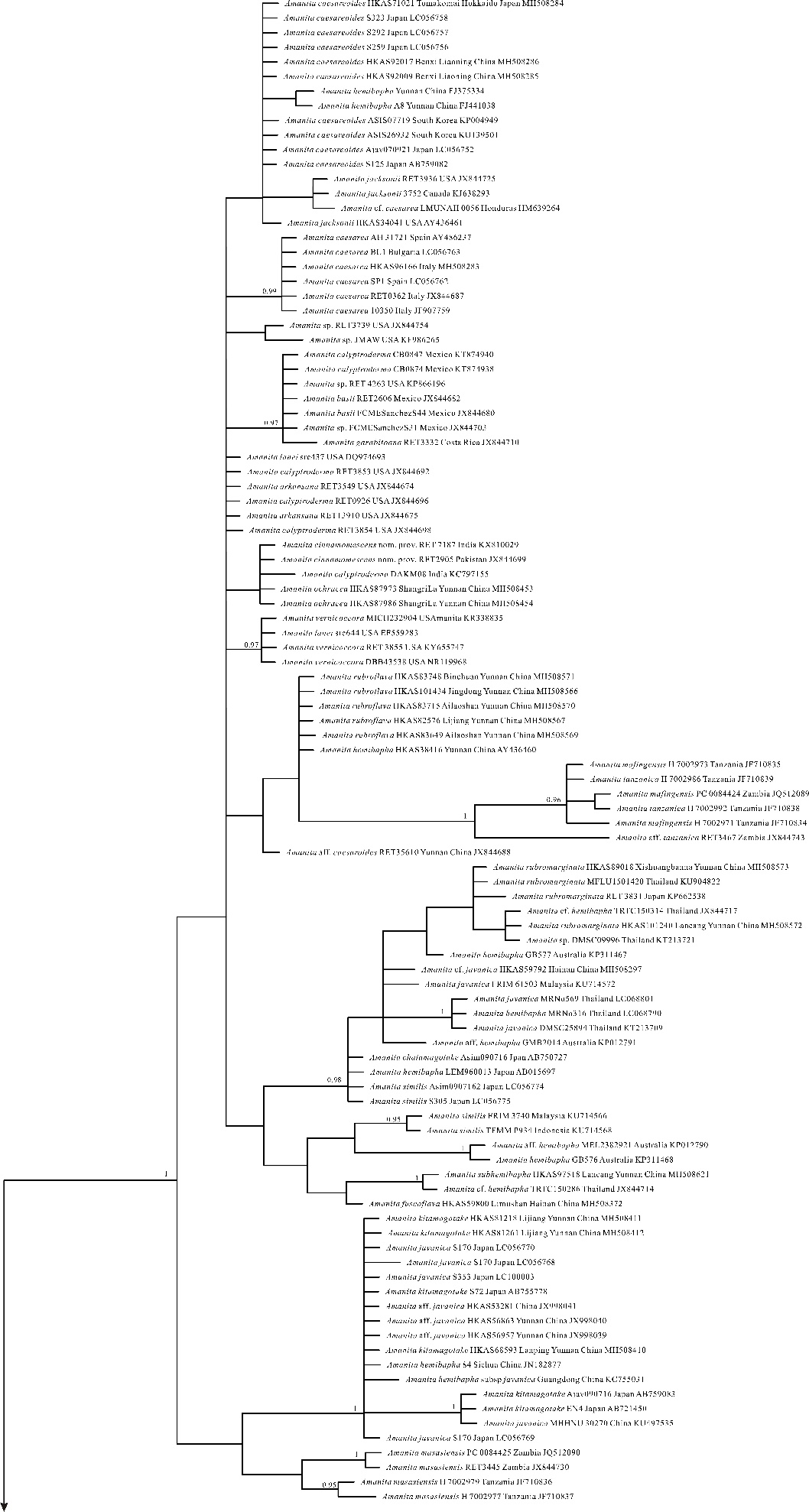


**Supplementary Figure S3 Phylogeny of *Amanita* sect. *Caesareae* inferred from the ITS sequences using the Bayesian analysis.**

Bayesian posterior probability (pp) values over 0.95 are shown above or beneath individual branches. New species are highlighted in red. Accession numbers of sequences information used are indicated on the Figure S3. The length of the aligned dataset was 492-bp long.


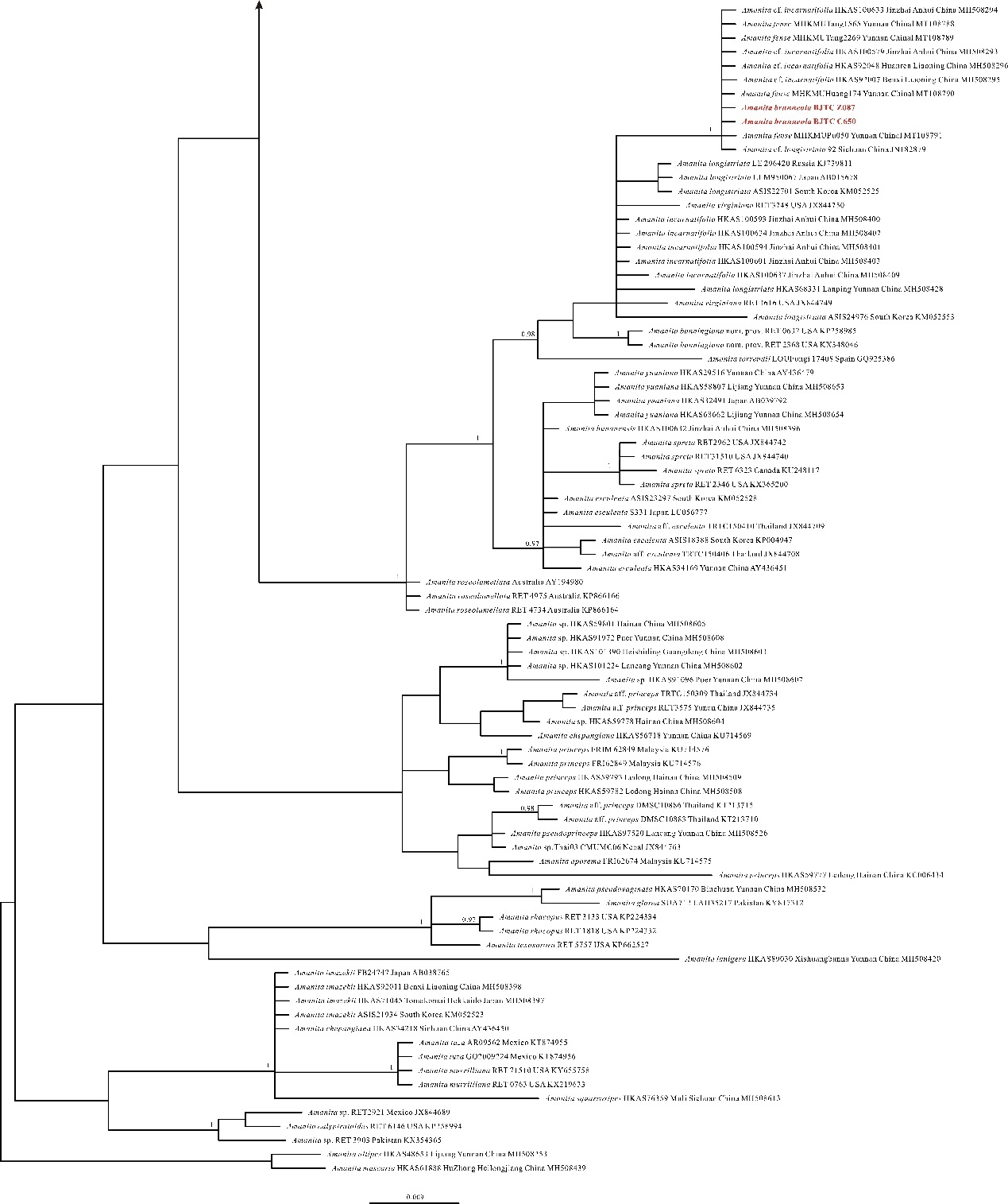


**Supplementary Figure S3 Continued**


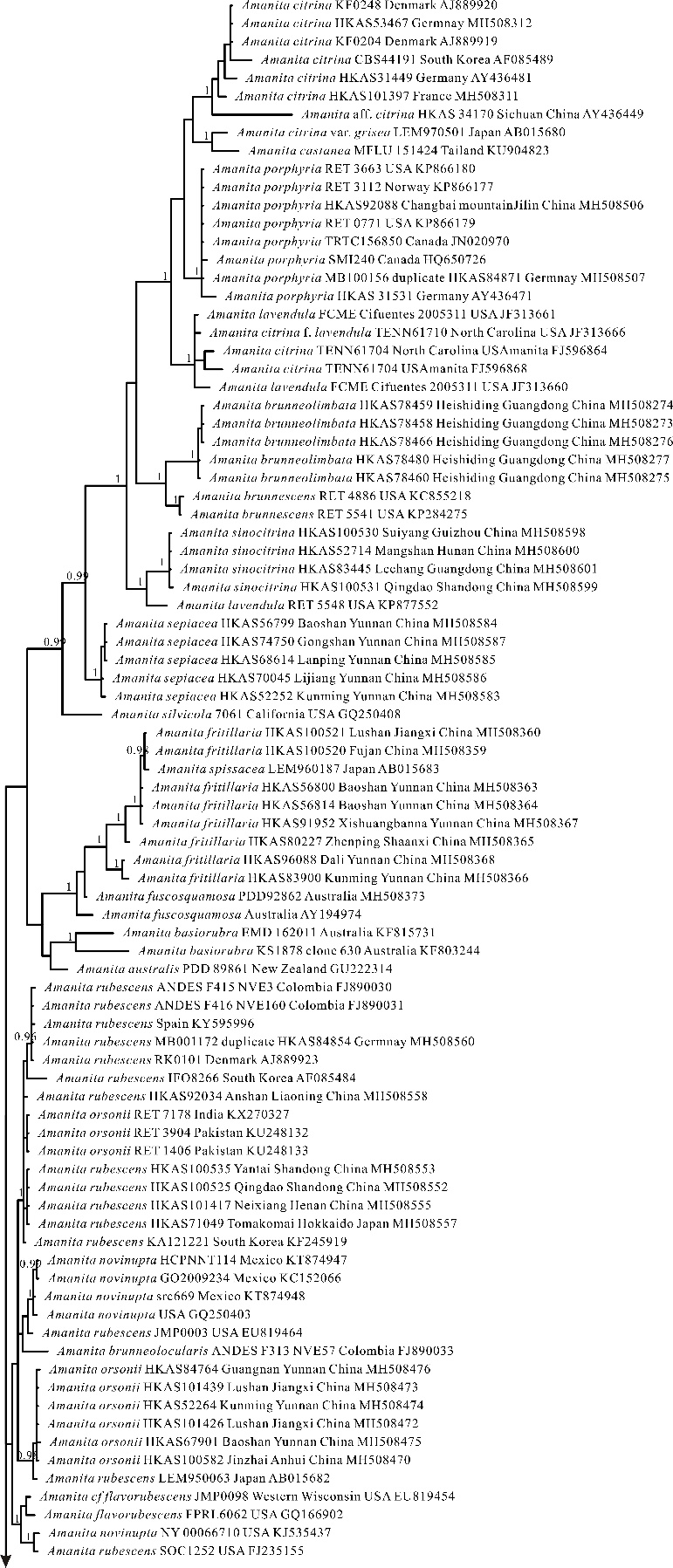


**Supplementary Figure S4 Phylogeny of *Amanita* sect. *Validae* inferred from the ITS sequences using the Bayesian analysis.**

Bayesian posterior probability (pp) values over 0.95 are shown above or beneath individual branches. New species are highlighted in red. Accession numbers of sequences information used are indicated on the Figure S4. The length of the aligned dataset was 540-bp long.


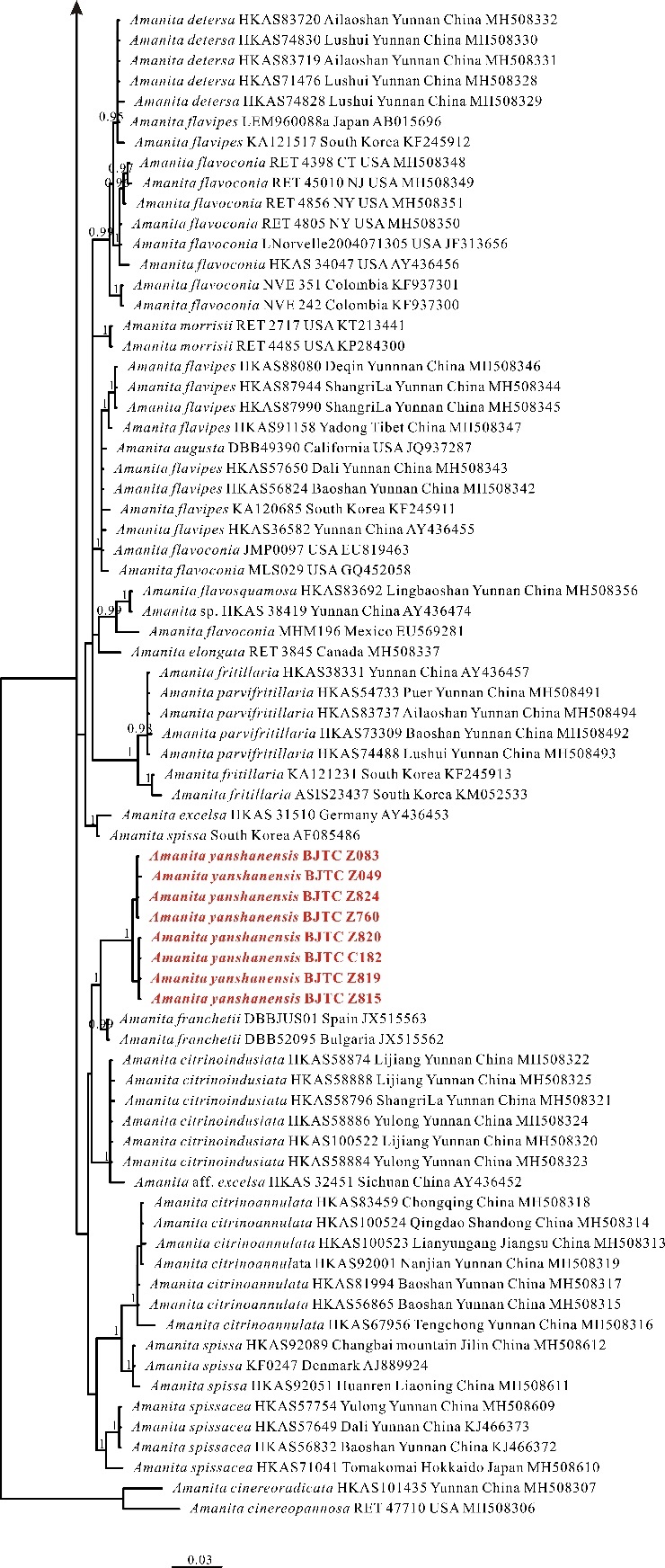


**Supplementary Figure S4 Continued**
